# A randomized feasibility trial of medium chain triglyceride-supplemented ketogenic diet in people with Parkinson's disease

**DOI:** 10.1186/s12883-024-03603-5

**Published:** 2024-04-01

**Authors:** Alexander H. Choi, Melanie Delgado, Kong Y. Chen, Stephanie T. Chung, Amber Courville, Sara A. Turner, Shanna Yang, Kayla Airaghi, Irene Dustin, Patrick McGurrin, Tianxia Wu, Mark Hallett, Debra J. Ehrlich

**Affiliations:** 1 National Institute of Neurological Disorders and Stroke, National Institutes of Health, Bethesda, MD USA; 2 Mid-Atlantic Permanente Medical Group, Kaiser Permanente Mid-Atlantic States, Rockville, MD USA; 3 National Institute of Diabetes and Digestive and Kidney Diseases, National Institutes of Health, Bethesda, MD USA; 4 NIH Clinical Center Nutrition Department, National Institutes of Health, Bethesda, MD USA

**Keywords:** Clinical trial, Parkinson’s disease, Ketogenic diet, Biomarker, Pilot study, Ketosis

## Abstract

**Background:**

A ketogenic diet (KD) may benefit people with neurodegenerative disorders marked by mitochondrial depolarization/insufficiency, including Parkinson’s disease (PD).

**Objective:**

Evaluate whether a KD supplemented by medium chain triglyceride (MCT-KD) oil is feasible and acceptable for PD patients. Furthermore, we explored the effects of MCT-KD on blood ketone levels, metabolic parameters, levodopa absorption, mobility, nonmotor symptoms, simple motor and cognitive tests, autonomic function, and resting-state electroencephalography (rsEEG).

**Methods:**

A one-week in-hospital, double-blind, randomized, placebo-controlled diet (MCT-KD vs. standard diet (SD)), followed by an at-home two-week open-label extension. The primary outcome was KD feasibility and acceptability. The secondary outcome was the change in Timed Up & Go (TUG) on day 7 of the diet intervention. Additional exploratory outcomes included the N-Back task, Unified Parkinson’s Disease Rating Scale, Non-Motor Symptom Scale, and rsEEG connectivity.

**Results:**

A total of 15/16 subjects completed the study. The mean acceptability was 2.3/3, indicating willingness to continue the KD. Day 7 TUG time was not significantly different between the SD and KD groups. The nonmotor symptom severity score was reduced at the week 3 visit and to a greater extent in the KD group. UPDRS, 3-back, and rsEEG measures were not significantly different between groups. Blood ketosis was attained by day 4 in the KD group and to a greater extent at week 3 than in the SD group. The plasma levodopa metabolites DOPAC and dopamine both showed nonsignificant increasing trends over 3 days in the KD vs. SD groups.

**Conclusions:**

An MCT-supplemented KD is feasible and acceptable to PD patients but requires further study to understand its effects on symptoms and disease.

**Trial Registration:**

Trial Registration Number NCT04584346, registration dates were Oct 14, 2020 – Sept 13, 2022.

**Supplementary Information:**

The online version contains supplementary material available at 10.1186/s12883-024-03603-5.

## Introduction

Parkinson’s disease (PD) is characterized by core deficits that include bradykinesia, rigidity, postural instability, and rest tremor. In addition to motor symptoms, nonmotor symptoms may also manifest. These include memory deficits, neuropsychiatric symptoms, and autonomic dysfunction, which often contribute to morbidity [1]. To date, available therapies treat only the symptoms, and disease-modifying therapies remain lacking.

Converging evidence implicates mitochondrial insufficiency in the pathogenesis of PD [2–5]. A common pathophysiological state underlying these deficits consistently found in PD is bioenergetic failure from deficient/faulty mitochondria, resultant oxidative stress, and toxic accumulation of predominantly oxidized membrane lipid species such as 4-hydroxynonenal with impaired autophagy. In PD, mitochondrial DNA damage mediated by ferric (Fe2 +)-induced hydroxyl radical production is found particularly in the substantia nigra and other highly metabolic, thinly myelinated neuronal populations. Laser capture of substantia nigra dopaminergic neurons has shown that peroxisome proliferator-activated receptor gamma coactivator 1-alpha** (**PGC1α) is downregulated and mitochondrial complex I in the Krebs cycle is inhibited [6]. Dendritic arborization is reduced with impaired punctate mitochondria in the striatum, with greater loss of terminals than cell bodies [7]. This suggests an opportunity for metabolic intervention to improve symptoms and potentially slow disease progression.

A growing body of literature supports a ketogenic diet (KD) for the treatment of neurodegenerative disorders marked by mitochondrial depolarization, calcium overload and energy failure, with reduced glucose uptake and impaired autophagic-lysosomal clearance of cellular waste [8–19]. Such disorders include Alzheimer’s disease (AD)/mild cognitive impairment-AD and PD. Ketosis achieved by reduced carbohydrate intake with or without fasting or increased physical activity produces an elevated primary circulating ketone body, d-betahydroxybutyrate (BHB), and an array of adaptive metabolic changes that are thought to be beneficial to the aging process and neurodegenerative diseases involving bioenergetic deficiency [20–24].

While there is enthusiasm for using a KD in neurodegenerative diseases such as PD, previous work has reported a significant challenge regarding tolerability, with study dropout rates of 14–28% [25–27]. Prior studies that evaluated a traditional KD in PD showed reduced total motor (Vanitallie et al. 2005) [25] and nonmotor scores (Phillips et al. 2018) [26] on the Unified Parkinson’s Disease Rating Scale (UPDRS) and improved cognition (Krikorian et al. 2019) [27]. However, the three clinical trial interventions to date were relatively labor intensive for study investigators, as well as subjects and their caretakers. These interventions involved weekly check-ins and daily meal preparations, the feasibility of which may be questionable on a larger scale.

The use of medium chain triglyceride (MCT) oil to supplement nutritional ketogenesis may offer several advantages over previous KD interventions. MCT oil reduces keto-induction time and augments mean levels of BHB compared to sunflower oil [28]. MCT supplementation of 30 mL three times daily supplies 630 kcal, approximately 30% of a typical person’s daily energy requirement and up to 50% of daily lipids, allowing for less radical dietary changes when compared to a traditional KD. Furthermore, MCT oil offers the advantage of rapid bioavailability to the liver and conversion to ketone bodies through three enzymatic steps. This accelerates the onset of ketogenesis and consequently reduces the period of symptomatic keto-induction. It also increases the concentration of ketone bodies in healthy volunteers for at least 8–10 h, and a 0.5–0.7 mM increase from last ingestion to morning fasting levels was observed [28]. Finally, MCT supplementation is amenable to placebo control and double blinding, thus permitting an intervention of the level of nutritional ketosis for study.

The goal of this pilot study was twofold: to determine if an MCT-supplemented KD would be feasible in a PD cohort and to explore potential ketosis biomarkers and symptomatic measures that may inform future study design. We hypothesized that acute ketosis with MCT supplementation studied over 3 weeks in a clinically moderate motor severity PD cohort would be feasible. We defined feasibility as a composite outcome, defined by meeting all of the following: average net carbohydrates < 10%; acceptability of ≥ 2/3 on a 0–3 Likert scale at week 3, meaning somewhat to very likely to continue a ketogenic diet such as tested in this study in the future; and study retention > 80%. To examine this hypothesis, we conducted a pilot clinical trial with a 1-week randomized double-blind comparison of a KD supplemented with MCT oil to a standard diet (SD), followed by a 2-week open-label phase. Baseline and end of study (week 3) exercise level defined by metabolic equivalent minutes per week was estimated by survey and maintained during the inpatient week to prevent deconditioning and confounding effect from loss of baseline exercise. The degree of ketosis as per blood concentration was measured as an exploratory outcome rather than a feasibility endpoint due to the limited literature of ketosis in Parkinson’s of varying study diet composition and infrequent data capture in the prior studies. However, the convention of nutritional ketosis as defined by blood concentration > 0.5 mM [29] was used as a confirmatory marker. Additionally, we tested the hypothesis that acute ketosis would improve mobility on the Timed Up & Go (TUG) test to a clinically meaningful extent on day 7 of diet intervention. The study duration and TUG were selected based on a hypothesis that acute ketosis may provide symptomatic motor improvement by lowering the production of mitochondrial reactive oxygen species and stimulating mitochondrial biogenesis, potentially inhibiting glutamatergic hyperexcitability in the striatum [30], which is implicated in the motor cortex-subthalamic hypersynchrony that corresponds to motor parkinsonism [31].

Separate from feasibility, additional exploratory markers, including blood concentration of ketones, levodopa, brain-derived neurotrophic factor (BDNF) and rsEEG, were investigated for the effect of ketosis or potential value as a biomarker of ketosis. Exploratory outcomes were chosen for novelty and to gauge value for further study, as, for instance, there is no prior literature to date on the effect of ketosis on levodopa absorption or gastrointestinal symptoms.

## Methods

### Study design

**Fig. 1:**
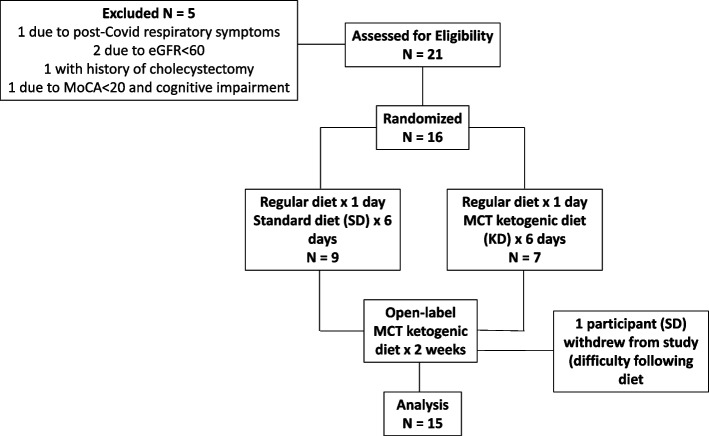
Flow chart for the one-week in-hospital, double-blind, randomized, placebo-controlled diet (MCT-supplemented ketogenic diet vs. standard diet), followed by an at-home two-week open-label extension in a cohort of patients with Parkinson’s disease

A pilot feasibility trial was conducted at the National Institutes of Health (NIH) Clinical Center. The study was approved by the NIH Institutional Review Board, and all subjects provided written informed consent prior to study enrollment. The study was conducted in two parts: a 1-week double-blind 1:1 randomized inpatient phase with daily assessments and a 2-week open-label outpatient phase (Fig. 1). The primary objective was feasibility, which was defined as successfully meeting all of the following criteria: (a) reaching recruitment target (*n* = 32, or *n* = 16 per pre-specified interim analysis); (b) retention rate above 80%; (c) adherence in the outpatient segment defined as average participant net (total—fiber) carbohydrate intake < 10% daily caloric total; and (d) acceptability defined via average exit survey at week 3 from a Likert scale (0–3) 2 or greater indicating at least somewhat likely to use a KD in the future. A prespecified interim analysis for futility was conducted at 50% enrollment (16 subjects). The secondary objective was to assess whether ketosis established over the study duration had a significant effect on mobility defined by the mean difference between KD and SD in TUG time at inpatient day 7. All other study measures were exploratory to assess symptoms, rating scales (NMSS, UPDRS and GSRS), and biomarkers of PD in a double-blinded fashion for 7 days and subsequently in an open-label fashion at week 3. Measures were selected with inclusion of NINDS Common Data Elements with goal to provide assessment of physiologic, cognitive, Parkinson symptom, autonomic, mood, GI, and potential biomarker of ketosis (EEG, BDNF).

During the 1-week inpatient phase, to control for approximate baseline exercise levels that varied between participants and to prevent deconditioning while in the hospital, each subject’s baseline exercise level was obtained from survey and interview data at screening and admission intakes and then approximately maintained using a stationary bike according to weekly metabolic equivalent x minutes. Subjects were asked to pedal at a comfortable, moderate intensity level. Some subjects, due to pain or prior routine/preference, used alternative exercise routines, which included walking around the unit/hospital, online guided exercises, or calisthenics. Exercise sessions were supervised and recorded by nursing staff. Exercise levels during the 2 weeks at home phase were recorded by directed recall during the week 3 visit.

#### Subjects

Subjects were recruited from the NIH Parkinson’s Disease Clinic or self-referral (Table 1).

**Table 1:** Subjects at screening visit, mean ± standard deviation or tally

	**Ketogenic Diet (*n* = 7)**	**Standard Diet (*n* = 9)**
Age (years)	67.3 ± 7.5	67.1 ± 4.5
Sex Female	2 (28.6%)	5 (55.6%)
White	6 (85.7%)	7 (77.8%)
Asian	1 (14.3%)	1 (11.1%)
Race not reported		1 (11.1%)
Years since PD diagnosis	15.2 ± 9	10.3 ± 5.3
LEDD (mg)	655 ± 340	738 ± 306
Bilateral STN	2 (28.6%)	4 (44.4%)
H&Y	2.5 ± 0.4	2.4 ± 0.4
NMSS	48 ± 19	44 ± 18
Waist circumference (cm)	91.4 ± 13.7	100.4 ± 9.3
Blood glucose (mg/dL)	92.6 ± 9.4	93.1 ± 10.3
Insulin (uIU/L)	8.8 ± 5.2	10.6 ± 4.2
Triglyceride (mg/dL)	82.2 ± 24.4	109.6 ± 35.2
HDL (mg/dL)	61.7 ± 16	46.3 ± 17.0
Metabolic syndrome	4 (57%)	2 (22%)
BMI (kg/m^2^)	29.5 ± 5.2	25.7 ± 2.8
Baseline carb %	54%	44%

Metabolic syndrome: Six subjects met the criteria for metabolic syndrome according to the AHA/NHLBI criteria (2004) and/or insulin resistance according to the threshold homeostasis model assessment of insulin resistance (HOMA-IR) > 2.0 [32].

*Bilateral STN* Received deep brain stimulation of bilateral subthalamic nucleus, *H&Y* Hoehn and Yahr Scale score, *LEDD* Daily Levodopa equivalent dose, *NMSS* MDS Non-Motor Symptom Scale.

Subjects were examined at a screening visit in 2020–2021 by a board-certified neurologist with focused motor examination for PD, UPDRS, and video recording and enrolled upon assessment by study PI. Subjects were eligible if they were fluent in English, able and willing to provide informed consent, age > 50 years with clinically probable diagnosis of PD using the UK Brain Bank Criteria, of moderate severity (Hoehn & Yahr Stage 2–4), with Montreal Cognitive Assessment (MoCA) ≥ 21, and ability to safely walk independently (with or without assistive device) for at least a short distance (20 feet) as determined during the screening visit. Exclusion criteria included diagnosis with another neurodegenerative disorder or significant kidney disease, liver disease, and uncontrolled diabetes or hyperlipidemia. A full description of the inclusion/exclusion criteria is listed online [33]. Of 21 screened subjects, 16 were enrolled (5 were excluded, including 1 due to post-COVID respiratory symptoms, 2 with eGFR < 60, 1 with a history of prior cholecystectomy, and 1 due to MoCA < 21 and cognitive impairment considered to interfere with study participation) (MCT-PD study, Fig. 1). All testing was performed in the medication ‘ON’ state, with dopaminergic and other medications maintained the same as the screening visit throughout the study, and effort was made to maintain consistent testing times in relation to dopaminergic medication doses.

### Primary outcome: feasibility and acceptability

During a 7-day inpatient admission at the NIH, baseline testing was performed on day 1, at which time subjects did not receive a controlled diet. On days 2–7, subjects received a randomized SD or KD. Subjects and clinicians performing the study procedures and rating scales were blinded to the diet group, while only nutrition staff were unblinded to diet randomization. Both the SD and KD were isocaloric. Each participant’s resting energy expenditure (REE) was calculated from the Weir formula [34]: metabolic rate (kcal per day) = 1.44 (3.94 V̇O2 + 1.11 V̇CO2) then multiplied by an activity factor of 1.6 to provide recommended daily calorie needs. The SD provided 35% energy from fat, 10–15% energy from protein, and 50–55% energy from carbohydrate. The KD provided 80% energy from fat, including 25% of daily fat from MCT oil, 10–15% energy from protein, and 5–10% energy from net carbohydrate, up to 20–50 g. Liquigen® (Nutricia) was used as the source of MCT oil in the KD diet and was incorporated into both foods and beverages. MCT oil meets the U.S. Food and Drug Administration’s generally recognized as safe (GRAS) exemption (21 CFR 182.1).

All meals (breakfast, lunch, dinner) for both the SD and KD groups consisted of one shake (either KD or SD) and one ketogenic snack for all participants to ensure double blinding, with methods and preparation similar to a prior study [35]. The meals were provided from the Nutrition Department’s Metabolic Kitchen with all foods and beverages weighed on a gram scale. Subjects were instructed to consume everything that was provided, and any refuse was weighed back. The integrity of subject blinding to dietary group was assessed on inpatient days 5–7 by asking each subject which type of diet they thought they were receiving.

Immediately following completion of the 1-week double-blinded inpatient phase, all subjects (SD and KD) were asked to follow a 2-week open-label MCT-supplemented KD at home. This diet was intended to meet the same macronutrient goals as the inpatient KD. Prior to inpatient discharge, subjects received education and instructions from a Registered Dietitian Nutritionist (RDN) on following the KD diet at home. Based on the subject’s individual calorie needs, RDNs prescribed a targeted number of 500 cal meals and 250 cal snacks from a KD cookbook specifically developed for this study. This cookbook included recipes with 2 tbsp (30 mL) of MCT oil (Liquigen) per meal and 4 tsp (20 mL) of MCT oil per snack [A table of contents of the home diet cookbook is available in a supplemental file– see Additional Table 1]. At the time of completion of the 1-week inpatient phase, subjects were provided with enough Liquigen for the 2-week outpatient diet phase.

Dietary intake and adherence to the MCT-supplemented KD were assessed by 24-h dietary recall, which were unannounced in order to avoid reactivity that can be associated with prospective dietary assessment methods. Recalls were administered by telephone interview by trained nutrition staff, who entered data directly into the Nutrition Data Systems for Research (NDSR) 2020. Recalls were administered on weekdays but aimed to capture intake on a variety of weekdays and Sundays for each participant. Recalls to assess baseline diet were collected twice during the week prior to admission. During the open-label extension, recalls were collected twice per week (total of 4 recalls during open label extension).

An exit survey was administered to each subject during the week 3 visit, including a question with a Likert scale of 0–3 of how likely they would be to continue a KD and additional questions regarding which benefits and side effects they experienced, if any (Psytoolkit version 3.3.2) [36, 37].

### Secondary outcome: timed up & go

During the screening visit, daily during each of the 7 inpatient days, and at the week 3 visit, the TUG test was performed to evaluate mobility. This was calculated as the time required to rise from a seated position in a chair, walk 10 feet, and return to the chair and sit back down. Three attempts were performed, with the fastest of the three used for analysis.

### Exploratory outcomes

#### Anthropomorphic, metabolic and laboratory assessments

Weight was obtained during the screening visit, inpatient days 1 and 7, and week 3. Waist circumference (at the iliac crest margin), bioelectric impedance absorption (BIA), and spectroscopy (BIS) were performed by study nutritionists and staff in the metabolic unit during the screening and week 3 visits according to standard techniques (BIA: [38]; BIS: [39]). Patients were categorized as whether or not meeting metabolic syndrome per AHA/NHLBI criteria (2004) and/or insulin resistance according to the threshold homeostasis model assessment of insulin resistance (HOMA-IR) > 2.0. These category designations were determined based on literature showing greater metabolic benefits of a ketogenic diet including weight loss, glycemic and lipid effects in subjects with metabolic syndrome or diabetes [40].

Indirect calorimetry via respiratory gas exchange was performed at screening and week 3 visits in the morning fasting state to derive resting energy expenditure, minute ventilations of oxygen and carbon dioxide in milliliters (V̇O_2_, V̇CO_2_) and respiratory quotient (RQ, V̇CO_2_/V̇O_2_), as previously described [41].

Continuous glucose monitoring system (Freestyle Libre Pro System sensor, Abbott) was worn by subjects in the upper outer arm per device label during inpatient days 1–7.

Blood draws were performed at defined intervals twice daily: fasting at 7 am (inpatient days 1–7) and at 2 pm, 1–1.5 h after lunch (inpatient days 1–6). Plasma levels of the levodopa metabolites dopamine-(3 and 4) sulfate, dopamine, and 3,4-dihydroxyphenylacetic acid (DOPAC) were measured on inpatient days 1–3 and at week 3, and plasma levels of ketones (BHB and acetoacetate) were measured on inpatient days 1–7 and at week 3 (National Institute on Alcohol Abuse and Alcoholism, liquid chromatography). Serum BDNF was measured on inpatient days 1 and 7 and at week 3 (ELISA, Raybiotech). The protocol for centrifugation of all samples was at 4 degrees Celsius for 10 min at 10,000 rpm.

Plasma hemoglobin A1c (HbA1c), lipid panel, insulin, glucose, cortisol, apolipoproteins A1 and B, C-reactive protein (CRP), thyroid stimulating hormone (TSH), T3, and free T4 were measured on inpatient days 1 and 7 and week 3, except for HbA1c (screening, week 3) and lipid panel (screening, inpatient day 7, week 3). Electrolytes (BMP, magnesium) were checked daily on inpatient days 2–6 as a safety measure. Testing was performed in accordance with CLIA-certified NIH Clinical Center Core Laboratory.

#### Physiological and clinical assessments

Prior to starting dietary intervention, a clinical electrocardiogram (ECG) was performed to exclude clinically significant arrhythmia at screening or inpatient day 1.

Vital signs, including daily supine/standing blood pressures at screening visit, inpatient days 1–7 and at week 3, were obtained. The 2-min heart rate variability was measured on inpatient days 1–7 and at week 3.

rsEEG was performed with the goal of assessing exploratory biomarkers of ketosis, as per a prior study showing increases in posterior alpha and central beta beta spectral power in healthy volunteers who received a classic ketogenic diet (4:1 fat: protein + carb) for 14 days [42]. EEG exams were performed on inpatient days 1 and 7 and during week 3 for a period of 20–30 min. A 64-channel cap was used for each session, with a layout according to the 10–20 System and a mastoid reference (Brain Products GmbH, Gilching, Germany). Impedances were generally kept under 5 kOhm. EEG data were amplified, digitized at 1000 Hz, manually inspected for noise, drift/movement artifacts removed, and offline filtered using average re-reference. To monitor cardiac activity, ECG signals were recorded from two precordial electrodes (left sternal and auscultation-based point of maximal impulse). Subjects were asked to enter a relaxed, wakeful state while minimizing movements for 5 min with eyes closed and then 5 min with eyes open. This was followed by 1 min of deep breathing and 3 runs of the Valsalva maneuver. Subjects were asked to blow into a syringe for 15 s targeting 30–40 mmHg via plethysmography as tolerated (for a few subjects, light-headed discomfort limited recording to 2 or 1 runs). The following metrics were analyzed:

Spectral and connectivity analyses (quantitative EEG) were performed using Brainwave (Stam7883). The frequency content from recordings with eyes closed and eyes open was assessed with a focus on activity in the theta (4–8 Hz), alpha (8–13 Hz), and beta (13–30 Hz) frequency bands. The 64-electrode montage was analyzed for relative spectral power by region, including frontal (F5 F3 F1/Fz/F2 F4 F6), central (C5, C3, C1/Cz/C2, C4, C6), and parietal (P5 P3 P1/Pz/P2 P4 P6) regions. Functional connectivity was assessed globally using the weighted graph theory network analysis via ‘Brainwave’ function (Javascript), with settings of 4096 ms epochs, gain 1.0, sample frequency 512 Hz, low pass filter 0.5 Hz, and high pass filter 70 Hz. We extracted the phase locking index (pairwise, nonzero phase asymmetry ranging from 0 to 1) that derives the “edge” or connection values of electrodes. We also extracted the weighted/mean clustering coefficient, CW_r (0–1 value corresponding to the degree of triangular signal clustering), and minimum spanning tree (MST) metrics, including weighted kappa (scalar indicating centrality or hub distribution of connections) [43]. The connectivity analysis was of interest to explore possible biomarkers of diet, given prior literature reporting benefits on cognitive functions including verbal memory and ADAS-Cog [20].

Heart rate variability (HRV), collected during inpatient days and 7 and at week 3, was analyzed using Kubios Pro, manually selecting the ECG channel with the best defined QRS complex for R-R detection. Deep breathing was performed at a directed 6 cycles per minute × 1 min, producing 5 cycles of expiratory:inspiratory (E:I) ratio. From deep breathing and Valsalva maneuver recordings, HR max:HR min was assessed using a “Get Peaks” MATLAB function to calculate the averaged E:I ratio and Valsalva Ratio (VR), respectively, per run.

HRV was also assessed during inpatient days 1–7 and at week 3 using photoplethysmography. This was performed using an EliteHRV Corsense and smartphone app for 2 min with the participant seated and resting. Outcomes were time domain (HR min, max, root mean square standard deviation (RMSSD), successive differences of NN intervals (SDNN), and low/high frequency spectral power.

UPDRS total score was assessed during the screening and week 3 visits with UPDRS Part 2 scales both ‘ON’ and ‘OFF’ per interviewer review; the UPDRS Part 3 was performed daily in the ‘ON” state on inpatient days 1–7 and at week 3 by a board-certified neurologist.

Simple cognitive tests were conducted daily during the inpatient phase and at the week 3 visit. Cognitive tests included simple and complex reaction time (simple instruction to press space bar to cue on fixation; complex instruction to press one of four keys, ‘z x <  > ’ upon corresponding visual cue); Stroop congruent and incongruent reaction times/error %, 3-back latency/error% (Psytoolkit). Motor tests included the MN and PQ keyboard tasks (30 s of alternating ‘M’ and ‘N’ key with each hand using digits 2 and 3, and time to complete 15 ‘P’ and ‘Q’ key strikes with index finger, each as quickly as possible (Presentation software), and the 9-hole pegboard test.

Administered surveys included (a) the Non-Motor Symptom Scale (NMSS, a 30-item scale encompassing a comprehensive inventory of PD-related nonmotor symptoms frequency x severity across 9 symptom domains (screening, week 3) [44]); (b) the Geriatric Depression scale (GDS-15) (screening, week 3); (c) the Gastrointestinal Symptom Rating Scale (GSRS) (screening, inpatient day 7, week 3) [45]; (d) a keto-induction survey using a 0–3 Likert scale for various symptoms related to metabolic shift to ketosis (inpatient days 1–7, week 3, Psytoolkit; adapted from Harvey et al. [28]) (Table 2); (e) a PD symptom survey using the Likert scale 0–3 for various daily PD symptoms not applicable, better than normal, normal, or worse than normal (inpatient days 1–7, week 3, Psytoolkit) (Table 3); and (f) the Gastrointestinal Symptom Diary: (International Foundation for Gastrointestinal Disorders, inpatient days 1–7, week 3) [46]; (g) the Exit survey, as described under Primary outcome above; and (h) a poststudy safety questionnaire (2–4 weeks after week 3).

**Table 2:** Keto-induction symptom survey administered on inpatient days 1–7 and week 3 outpatient visit

Symptom	Score
Difficulty concentrating	
New rash	
Felt weaker than normal	
Had muscle cramps	
Had halitosis	
Had abdominal pain	
Had diarrhea	
Had constipation	
Had headache	

In the past 24 h (select one of the following):

0 = No.

1 = Yes.

**Table 3:** Administered Parkinson symptom survey on inpatient days 1–7 and week 3

Symptom						Score
**The following applies to my tremor:**						
**The following applies to my dyskinesia:**						
**The following applies to my daily off time:**						
**The following applies to my concentration:**						
**The following applies to my urinary / urinary urge frequency:**						
**The following applies to my pain:**						
**I have had slow movements:**						
**I have felt depressed:**						
**I have felt anxious:**						
**I have felt fatigue:**						
**I have felt unmotivated or lack of desire for my daily activities:**						
**I have had one or more episodes of dystonia (such as toe or finger curling or twisting):**						
**How much (percentage) of the waking day yesterday did you feel off…**						
**Did you feel ‘off’? (wearing off or lack of response to Parkinson medication)**						**Score**
0%	1–10%	11–25%	26–50%	51–75%	76% or greater	
1	2	3	4	5	6	

In the past 24 h (select one of the following):

1. I feel better than normal
2. I feel normal
3. I feel worse than normal
4. Not Applicable

### Statistics

#### Power analysis

A power analysis was performed based on the TUG score at inpatient day 7 (the secondary outcome) since the feasibility outcome (the primary outcome) did not involve a statistical hypothesis. According to previous studies, the TUG score in PD patients off dopaminergic medication had a range of 8–17 s [47], a mean of 10–15 s, and a standard deviation of 2.15–3.7 s [47–50]. Minimal detectable changes were reported as 4.85 s [49] and 3.5 s [50], and a 1-s increase in TUG was associated with a 5.4% increase in the relative risk of reported falls [51]. Therefore, the difference of 4.5 s between the group means with a standard deviation (SD) of 4 s was considered clinically important and therefore used for the power analysis.

A two-sample t test was applied to estimate sample size by assuming that the TUG was normally distributed and that the variances of the two groups were equal. The power analysis indicated that a total of 28 subjects, 14 per group, were needed to detect a difference of 4.5 s at a significance level (α) of 0.05 and a power of 80%. By anticipating a 10% dropout rate, a total of 32 subjects (16 per group) would be included in the randomization. An interim analysis for futility based on the TUG effect was scheduled to be conducted when 16 subjects had completed the study. Using the O'Brien‒Fleming method, α = 0.0054 was applied in the interim analysis, and α = 0.05 was applied in the final analysis.

#### Statistical analysis methods

Analysis of covariance (ANCOVA) was used to evaluate the diet effect on TUG at inpatient day 7 with age and TUG at baseline as covariates. Either a repeated measures analysis of variance (RM-ANOVA) or covariance (RM-ANCOVA, age as a covariate) was performed to examine the diet effect on the other metabolic variables, which included BIA (resistance, reactance), height, weight, waist circumference, and BIS (fat free mass, body fat %, skeletal muscle mass, total body water, and visceral fat). The RM-ANOVA model included diet group (KD vs. SD), time (baseline vs. week 3), and the interaction between time and group. For the above variables with no significant diet effect, either a one-sample t test or Wilcoxon signed rank test was applied to evaluate the change from baseline to week 3. Other comparisons were made between the KD and SD groups with two-sample, two-tailed t tests.

**Fig. 2:**
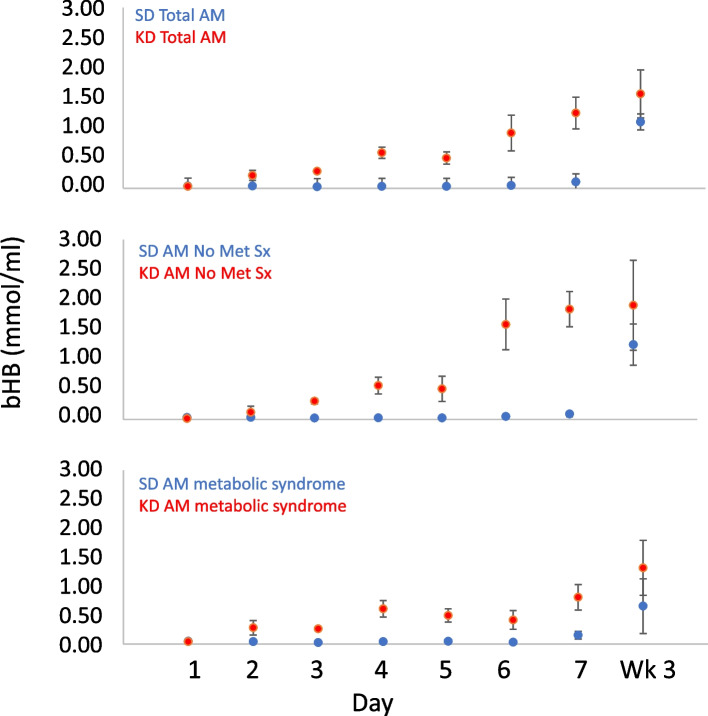
Reported AM fasting beta-hydroxybutyrate (BHB) mean values ± SEMs, days 1–7 (inpatient) and at week 3 (outpatient), by group. Day 1: baseline testing on regular diet; Days 2-7: inpatient randomized diet phase-ketogenic diet vs standard diet; Week 3: Assessment after 2 weeks of outpatient open-label ketogenic diet. Top: Total 7 subjects in the KD group vs. 9 subjects in the SD group; Middle: 3 subjects in the KD group vs. 7 subjects in the SD group without metabolic syndrome/insulin resistance; Bottom: 4 subjects in the KD group vs. 2 subjects in the SD group with metabolic syndrome/insulin resistance

All statistical analyses were performed using SAS version 9.4 or SPSS, and α = 0.05 was used as the significance level since all tests, except for the TUG test at inpatient day 7, were exploratory. The diet group means, standard deviations, and tallies are presented in Fig. 2 and Table 4–15. CONSORT reporting guidelines were adhered to [52].

**Table 4:** Nutrient intake from 24-h dietary recalls

	Pre- intervention KD group	Post-intervention KDgroup	Pre-intervention SD group	Post-intervention SD group
Energy (kcal)	1893 (397)	2139 (490)	1695 (536)	1857 (367)
Total Fat (g), %	72 (22), 34%	185 (41), 78%	75 (32), 40%	161 (42), 78%
Total Carb (g), %	255 (69), 54%	60 (17), 11%	186 (52), 44%	61 (17), 13%
Net Carb %	49%	9%	39%	10%
Total Protein (g)	65 (24), 14%	67 (33), 13%	60 (16), 14%	49 (12), 11%
Fructose (g)	23 (13)	5 (4)	15 (12)	5 (2)
Sucrose (g)	60 (34)	5 (5)	29 (20)	7 (5)
Starch (g)	93 (40)	14 (7)	83 (26)	11 (10)
Dietary Fiber (g)	21 (8)	13 (5)	20 (9)	14 (5)
Sodium (mg)	2483 (820)	2406 (1176)	2331 (910)	1909 (784)
Potassium (mg)	2667 (1040)	1924 (572)	2306 (719)	1991 (513)
SFA 8:0 (caprylic acid) (g)	0.6 (0.3)	38 (10)	0.6 (0.8)	31 (10)

Mean (standard deviation) for the baseline (pre-intervention, 2 recalls/1 week) and week 3 (post ketosis intervention, 2–4 recalls (mean 3.5)*/2 weeks) for the ketogenic (KD) and standard diet (SD) groups.

*Some participants had 2 or 3 recalls only for the 2 weeks of ketogenic diet at home due to missing data.

#### Randomization

The stratified block randomization method was used to control and balance the covariates of age (≤ 65 vs. > 65 years) and TUG baseline time (≥ 11.5 s, vs. < 11.5 s), given that TUG time is found to vary by age and by baseline TUG time [48], as well as to ensure a balance in sample size across groups over time. First, the subjects were assigned to one of the 4 possible strata: TUG < 11.5 s & age ≤ 65 years, TUG < 11.5 s & age > 65 years, TUG ≥ 11.5 s & age ≤ 65 years, TUG ≥ 11.5 s & age > 65 years. Then, subjects were assigned to receive a KD or SD using block randomization (block size = 4) by study statistician and assigned to KD or SD intervention by study dieticians.

## Results

### Primary outcome: feasibility

A total of 15/16 subjects completed the study (6 female, mean age 67 years, BMI 27.6 kg/m^2^, Hoehn & Yahr stage 2–3), 6 with metabolic syndrome and/or insulin resistance.

One subject withdrew after completing the inpatient 7-day blinded diet intervention due to difficulty following the KD at home, permitting interim analysis of TUG with all 16 subjects. The results reported are as per prespecified interim analysis of the 16 participants by group per protocol. The mean baseline macronutrient percentages were as follows: KD group total carbohydrate 54%, total fat 34%, total protein 14%; SD group total carbohydrate 44%, total fat 40%, total protein 14% (Table 4).

There were no reported differences in keto induction symptoms over the inpatient week (Table 5).

**Table 5:** Daily keto-induction symptom log

	Ketogenic Diet								Standard Diet							
Visit	Day 1	Day 2	Day 3	Day 4	Day 5	Day 6	Day 7	Wk 3	Day 1	Day 2	Day 3	Day 4	Day 5	Day 6	Day 7	Wk 3
concentration	0	0	0	0	0	0	0	1	0	0	0	0	0	0	0	0
rash	0	0	0	0	0	0	0	1	0	0	0	0	0	0	0	0
weakness	1	0	0	0	2	1	0	0	0	0	1	0	0	0	1	2
cramp	2	2	3	1	1	3	1	1	1	1	2	2	2	2	3	2
halitosis	1	1	1	1	1	2	1	0	0	0	0	0	0	0	0	1
abdominal cramp	0	0	1	1	1	2	0	1	0	0	2	3	2	1	0	0
diarrhea	0	0	0	0	0	0	0	0	0	1	0	1	0	0	0	0
constipation	2	2	3	3	3	2	0	4	1	3	3	1	1	3	4	2
headache	0	2	1	3	3	1	1	2	1	1	4	4	1	1	3	2

Daily keto-induction symptom log, Ketogenic Diet (KD) and Standard Diet (SD) for inpatient days 1–7 and Week 3. Listed by row, number of subjects reporting the symptom over the preceding 24 h.

The daily PD symptom survey showed similar PD symptoms between the KD and SD groups, with trends toward less pain and urinary frequency in the SD group; however, there were no significant differences aside from greater daily percent off time in the SD group (*p* = 0.0005, Table 6).

**Table 6:** Daily Parkinson symptom log

	Ketogenic Diet								Standard Diet							
Visit	Day 1	Day 2	Day 3	Day 4	Day 5	Day 6	Day 7	Wk 3	Day 1	Day 2	Day 3	Day 4	Day 5	Day 6	Day 7	Wk 3
tremor	1	1.17	1	1	1	1	1	1	1	0.6	1	1	0.8	1.2	1	0.83
dyskinesia	1.25	1.25	1	1	0.75	1	1	0.67	1	1	1.4	1.2	0.8	0.8	1.17	0.67
off medication state	1.25	1.2	1	1	1	1	1	0.8	1.14	1	1	1	1	1.13	0.86	0.86
concentration	1	1	1	1	1.33	1	1		1	1	1	1	1	1	1	1
pain	0.8	0.8	1.2	1.4	1	1	1	0.67	1.17	0.67	1	0.67	1	0.71	0.83	1
urinary frequency	1	0.83	1	1	1.17	1	1.25	0.67	1	1.13	1	0.89	0.78	0.88	0.78	0.71
bradykinesia	1	1.17	1	1	0.83	0.75	1.2	1	1	1	1	1	1	0.78	1	0.67
depression	1	1	1.5	1	1	1.5	0.5	1	1.33	1	0.67	0.67	1	1	1	1
anxiety	1	1	1	1	1	1	1	0.67	1	1.2	0.8	1.33	1	1	1	1
fatigue	1	0.8	1	0.75	1.2	1.8	1	1.2	1.17	1.33	1.43	0.88	1.17	1.14	1.17	1.17
apathy	1	1	1	1.33	1	1	1	1	1	1	1	0.67	1	1	1	1
dystonia	1.6	1	1	1	0.75	1	1	0.83	1	1	1	1	1	0.8	1	1.2
% off	0.5	0.67	0.83	0.5	0.5	0.67	0.5	0.83	1.22	0.78	0.89	1.11	1.11	1.11	1.22	1.29

Parkinson symptom daily inventory, administered on inpatient day 1 (regular diet), inpatient days 2–7 (randomized ketogenic diet (KD) or standard diet (SD), and final study visit (Week 3, following 2 weeks outpatient KD for both groups). Reported are mean values. Value < 1 indicates better than normal, and > 1 indicates worse than normal. Daily off time was greater in the SD group than KD group (*p* = 0.00005), otherwise group differences were not statistically significant.

*Dysk* Dyskinesia, *off* off medication state, *conc* concentration, *bradyk* bradykinesia, *depr* depression, *anx* anxiety.

•For PD symptoms: 0 is better than normal 1 is normal 2 is worse than normal.

•For (daily) % off (of medication state), 0 = 0%, 1 = 1–10%, 2 = 11–25% 3 = 26–50% 4 =  > 50%

Four of 7 subjects in the KD group correctly guessed their group allocation, and 0/9 in the SD group did so, with 13 of 16 subjects guessing they were in the KD group (most incorrectly).

The mean (SD) net carbohydrate intake over the 2-week at-home KD was 9.7 (3.1)% of energy, with 9/15 participants (3/6 women, 6/9 men) maintaining a mean net carbohydrate intake below 10% of energy intake. The net carbohydrate data per subject are listed in an additional file [see Additional Table 2].

On the exit survey during the week 3 visit, the mean Likert survey score was 2.26/3 in 15 study completers, with only 3/15 rating unlikely to very unlikely to use the KD in the future. Week 3 survey results are shown in an additional file [see Additional Tables 3–4]. The survey results indicate that the majority (12/15) of participants were somewhat likely to very likely to continue a similar KD in the future.

The most commonly reported benefits were increased energy (*n* = 6), improved motor function (*n* = 6), decreased appetite (*n* = 4), improved constipation (*n* = 4), and less fatigue (*n* = 4). Side effects were reported as fatigue (*n* = 5), light-headedness, headache, reduced energy, off symptoms of urinary incontinence and gait freezing (*n* = 1), diarrhea (*n* = 1) (Table 7):

**Table 7:** Benefits and side effects

Benefits	Side Effects
Increased energy (*n* = 6)	Fatigue (*n* = 5)
Reduced tremor/off time/rigidity/motor fluctuations (*n* = 4)	Light-headedness (*n* = 1)
Reduced fatigue (*n* = 5) ± increased subjective mental clarity	Headache (*n* = 1)
Reduced appetite (*n* = 4)	Reduced energy (*n* = 1)
Reduced constipation (*n* = 4)	Off symptoms—urinary incontinence, gait freezing (*n* = 1)
Reduced muscle cramps, not RLS (*n* = 3)	Diarrhea × 1 (*n* = 1)
Reduced urination (*n *= 2)	
Improved emotional status (*n* = 2)	
Improved balance (*n* = 2)	
Reduced insomnia/visual hallucinations (*n* = 2)	
Reduced dysphagia (*n* = 1)	

Reported benefits and side effects of a ketogenic diet at the week 3 visit.

Note: some participants reported more than one benefit, and tallies are therefore not mutually exclusive. For instance, increased energy and reduced appetite were reported together as benefits. Off time: wearing off dopaminergic medication.

Adverse events attributed to the KD included fatigue (*n* = 5), headache/light-headedness (*n* = 2), possible PD symptoms (new onset freezing of gait, increased urinary frequency (*n* = 1), difficulty preparing the diet (*n* = 2) and difficulty following the diet vs. spouse’s diet (*n* = 2). There was one serious adverse event, deep vein thrombosis/pulmonary embolus, which occurred in a participant in the KD group. This developed several days after hospital discharge, while on the home phase of KD, in a subject with previously diagnosed beta thalassemia minor. An independent medical monitor review determined that this was unlikely to be related to the dietary intervention (Table 8).

**Table 8:** Adverse events

	7 days inpatient		2 weeks at home
	Ketogenic Diet	Standard Diet	Ketogenic diet
Hypotension	-	1/9 (11%)	
Constipation	1/7 (14%)	-	2/15 (13%)
Diarrhea	-	1/9 (11%)	1/15 (7%)
Increased salivation	1/7 (14%)	-	-
Indigestion and/or acid reflux	2/7 (29%)	1/9 (11%)	-
Fatigue	1/7 (14%)	1/9 (11%)	5/15 (33%)
Muscle ache	1/7 (14%)	-	-
Lower back pain	1/7 (14%)	-	-
Musculoskeletal pain	1/7 (14%)	-	1/15 (7%)
Headache	-	1/9 (11%)	1/15 (7%)
Dyskinesia	-	1/9 (11%)	-
Malaise	-	-	1/15 (7%)
Dystonia (toe curling/external rotation)	-	-	1/15 (7%)
Nausea	-	-	1/15 (7%)
Pulmonary embolus	-	-	1/15 (7%)

### Secondary outcome: timed up & go

Mean TUG times were reduced in both groups when comparing inpatient day 1 (KD: 8.7 ± 1.1; SD: 10.2 ± 2.8) to inpatient day 7 (KD: 8.2 ± 1.0 s; SD: 9.1 ± 2.3 s). The times were similar at week 3 (KD: 8.3 ± 1.2 s, SD 9.6 ± 2.7 s). Adjusted for baseline TUG, there was no significant difference between groups on inpatient day 7 (KD 8.4 s, SD 9.1 s, *p* = 0.23) (Table 9).

**Table 9:** Timed Up & Go (TUG)

Visit	SD Best TUG (sd)	KD Best TUG (sd)
Screening	10.2 (2.8)	8.7 (1.1)
Day 1	9 (2.4)	9.1 (2.1)
Day 2	8.8 (1.9)	9.5 (1.8)
Day 3	9.1 (2.2)	8.8 (1.9)
Day 4	9.3 (2.2)	9.3 (2.0)
Day 5	9 (2.1)	8.7 (1.5)
Day 6	8.8 (2.2)	9 (1.7)
Day 7	9.1 (2.3)	8.4 (1.2)
Week 3	9.6 (2.7)	8.3 (1.2)

Mean and standard deviation (sd) values by group for the ketogenic (KD) and standard diet (SD) groups. TUG values were based on the fastest time of three runs.

The predetermined endpoint of a mean difference in TUG time on inpatient day 7 between the KD and SD groups in the interim futility analysis was not met; therefore, the study was terminated after 16 participants.

### Exploratory outcomes

Survey results of mean metabolic equivalents from physical activity and exercise were comparable and increased in both groups from baseline to week 3 (KD: 812 to 943, SD: 917 to 1145 metabolic equivalents x minutes per week).

Nonmotor severity scale scores (NMSS) significantly decreased for all subjects from baseline to the 3-week visit, 43.8 ± 17.6 to 31.5 ± 13.2 (two-tailed t test p value = 0.04). The change in the KD group was 46.1 ± 16.2 to 24.4 ± 12.7 (domains with 3/7 or more participants showing improvement by ≥ 2 points were gastrointestinal (GI), urinary, sex, and miscellaneous, and 3/7 or more worsening by ≥ 2 points was sleep/fatigue), and in the SD group was 43.8 ± 15.1 to 33 ± 10.0 (domains with 3/8 or more participants improving were GI, urinary, and memory/cognition, and 3/8 or more worsening were sleep/fatigue and miscellaneous). The GI symptom rating scale declined somewhat, indicating improvement in both groups (KD 11.6 ± 9.1 (screening), 7.6 ± 6.2 (inpatient day 7), and 4.4 ± 3.5 (week 3); SD 8.5 ± 5.4 (screening), 4.5 ± 3.7 (inpatient day 7), and 5.8 ± 4.6 (week 3)).

Blood ketones (BHB) increased to above 0.5 mM in the KD group by inpatient day 4 (0.59 ± 0.25 mM), with an attenuation of further increase on inpatient days 5–7 and at the week 3 visit in the 4 subjects with metabolic syndrome and/or insulin resistance compared to the 3 subjects without such syndrome (Fig. 2, Table 10).

**Table 10:** Plasma concentration of ketone bodies

	**AM**		**PM**	
**SD**	BHB	AcAc	BHB	AcAc
	mmol/mL	mmol/mL	mmol/mL	mmol/mL
**D1**	0.04 (0.03)	0.01 (0.01)	0.03 (0.02)	0.01 (0.01)
**D2**	0.04 (0.04)	0.01 (0.01)	0.02 (0.01)	0.01 (0.00)
**D3**	0.03 (0.02)	0.01 (0.01)	0.01 (0.01)	0.01 (0.00)
**D4**	0.03 (0.02)	0.01 (0.01)	0.02 (0.02)	0.01 (0.01)
**D5**	0.03 (0.02)	0.01 (0.01)	0.02 (0.01)	0.01 (0.00)
**D6**	0.05 (0.07)	0.02 (0.03)	0.03 (0.03)	0.01 (0.01)
**D7**	0.11 (0.11)	0.04 (0.04)		
**Wk3**	1.1 (0.85)	0.37 (0.28)		
	AM		PM	
**KD**	BHB	AcAc	BHB	AcAc
	mmol/mL	mmol/mL	mmol/mL	mmol/mL
**D1**	0.03 (0.04)	0.01 (0.02)	0.06 (0.08)	0.02 (0.03)
**D2**	0.21 (0.22)	0.06 (0.06)	0.24 (0.21)	0.08 (0.06)
**D3**	0.28 (0.07)	0.1 (0.03)	0.6 (0.29)	0.21 (0.13)
**D4**	0.59 (0.25)	0.23 (0.09)	1.05 (0.31)	0.39 (0.13)
**D5**	0.5 (0.27)	0.17 (0.08)	0.83 (0.31)	0.27 (0.10)
**D6**	0.92 (0.79)	0.3 (0.27)	1.21 (0.82)	0.42 (0.29)
**D7**	1.25 (0.70)	0.42 (0.24)		
**Wk3**	1.57 (1.05)	0.53 (0.31)		

Beta-hydroxybutyrate (BHB) acetoacetate (AcAc), mean (standard deviation). Top plot (SD) shows Standard Diet group and bottom plot shows Ketogenic Diet group. AM is fasting at 7am, and PM is post-prandial at 2PM.

The levodopa metabolite 3,4-sulfated dopamine did not show a clear effect of diet, whereas dopamine and DOPAC, while individually variable, showed a mean elevation from D1 to D2-D3 for dopamine and DOPAC metabolites in the KD group compared to the SD group; however, neither difference was significant (p value dopamine = 0.23, p value DOPAC = 0.06). Mean plasma dopamine levels were increased in the afternoon in both groups (Table 11).

**Table 11:** Daily levodopa metabolites in plasma, inpatient days 1–3 and week 3 SD and KD group, mean (standard deviation)

Standard Diet	Dopamine-(3 and 4) sulfate (pmol/mL)	Dopamine (pmol/mL)	DOPAC (pmol/mL)
Day 1 AM	31.07 (17.57)	0.88 (0.46)	12.07 (4.26)
Day 1 PM	25.41 (11.70)	0.92 (0.27)	13.44 (6.92)
Day 2 AM	41.35 (13.28)	0.55 (0.16)	9.11 (3.35)
Day 2 PM	24.36 (9.98)	1.10 (0.43)	14.21 (9.24)
Day 3 AM	26.23 (9.77)	0.96 (0.40)	16.88 (6.96)
Day 3 PM	45.63 (38.67)	1.22 (0.56)	29.48 (6.63)
Wk 3 AM	42.48 (31.63)	2.16 (2.29)	24.87 (13.70)
Keto Diet	Dopamine-(3 and 4) sulfate (pmol/mL)	Dopamine (pmol/mL)	DOPAC (pmol/mL)
Day 1 AM	31.33 (11.64)	1.08 (0.52)	10.17 (3.70)
Day 1 PM	28.07 (9.25)	1.18 (0.53)	11.23 (3.03)
Day 2 AM	44.46 (22.28)	0.62 (0.46)	22.28 (18.76)
Day 2 PM	27.92 (13.90)	1.69 (0.50)	16.44 (10.25)
Day 3 AM	15.79 (5.64)	1.02 (1.04)	32.16 (6.90)
Day 3 PM	26.92 (6.87)	2.13 (1.57)	25.85 (9.85)
Wk 3 AM	24.66 (7.75)	1.35 (1.10)	24.01 (16.50)

*DOPAC* = 3,4-Dihydroxyphenylacetic acid

Between screening and week 3, there were significant reductions in weight (2.6 ± 1.3 kg, p < 0.0001), waist circumference, and BIS attributed to skeletal muscle mass for both the SD and KD groups. These changes were not significantly different when comparing between groups, so these data were pooled for statistical comparison. The changes in these metrics per patient group were as follows: waist circumference reduction (2.5 ± 3.3 cm, p = 0.01) and reduction in mass attributed to ‘skeletal’ via BIS (2.6 ± 3.9 kg, p = 0.05). From screening to week 3, there were reductions in both groups in REE and V̇CO2 > V̇O2, with a reduction in the respiratory quotient (RQ) from 0.80 (0.05) to 0.73 (0.03) (p = 0.000003) (Table 12). There were unchanged to modestly increased reported exercise levels in both groups from baseline to week 3 visit ([see Additional Fig. 1]).

**Table 12:** Anthropomorphic and Respiratory Gas Exchange Measurements

	**Screening**	**Week 3**
Weight (kg)*	74.1 (14.2)	71.5 (13.6)
abdominal circumference (cm)	95.5 (12.9)	92.6 (13.4)
BIA-resistance (ohm)	527.5 (118.0)	536.3 (118.1)
BIA-reactance (ohm)	52.5 (11.4)	55.9 (12.7)
Fat Free Mass (kg)	50.3 (11.7)	50.7 (11.4)
Body fat %	32.1 (8.2)	31.3 (9.6)
Skeletal Mass (kg)*	25.2 (5.7)	22.6 (6.9)
Total Body Water	37.8 (8.8)	37.7 (8.3)
Visceral fat (kg)	3.9 (2.1)	3.5 (1.9)
REE (kcal)	1441.8 (241.4)	1328.6 (227.1)
VO2/min (L/min)	210.1 (34.4)	193.7 (34.9)
VO2/kg/min (L/kg*min)	2.90 (0.27)	2.73 (0.31)
VCO2/min (L/min)	168.8 (25.8)	132.8 (45.5)
RQ*	0.80 (0.05)	0.73 (0.03)

*P-value < 0.05

**Fig. 3:**
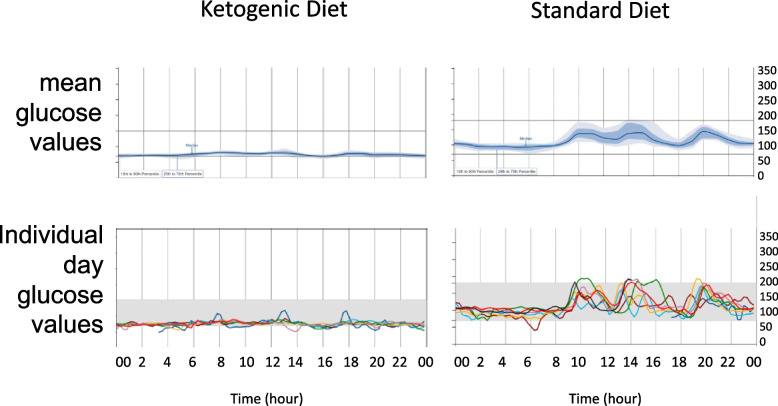
Continuous glucose monitor (CGM) results over 7 inpatient days for representative participants on a standard diet (top 2 plots, average/daily) and on a ketogenic diet (bottom 2 plots, average/daily). The top single line plot for each representative participant indicates mean glucose values, and the bottom multiple line plot shows each day’s values

Continuous glucose monitoring showed a clear difference between the KD and SD groups on each day during inpatient days 1–7, with each subject in the KD group showing a stable glucose level (60–140), whereas the SD group showed mealtime boluses for 2 h and baseline levels of 80–100 rising to 150–180 mg/dL with meals (Fig. 3). Insulin was reduced in the KD group (10.6 ± 4.2 pg/mL inpatient day 1; 6.9 ± 4.8; inpatient day 7; 8.1 ± 3.0 pg/mL week 3) and to a lesser extent in the SD group (9.3 ± 5.4 inpatient day 1; 8.4 ± 2.7 inpatient day 7; 8.2 ± 3.7 pg/mL week 3). Total triglycerides decreased and HDL increased in both groups over the study to week 3 but to a greater extent in the KD group than in the SD group. There were no statistically significant differences in CRP or HbA1c from the intervention in either group (excluding one subject with DVT/PE with spurious CRP elevation) (Table 13).

**Table 13:** Metabolic labs

	Ketogenic Diet			Standard Diet		
	Day 1	Day 7	Week 3	Day 1	Day 7	Week 3
Glucose (mg/dL)	96.4 (8.5)	88.9 (8.5)	90.8 (6.4)	94.9 (14.8)	91.3 (16.7)	90.7 (12.9)
Insulin (µIU/mL)	10.6 (4.2)	6.9 (4.8)	8.1 (3.0)	9.3 (5.4)	8.4 (2.7)	8.2 (3.7)
Total cholesterol (mg/dL)	158.3 (35.1)	148.4 (28.4)	153.4 (27.2)	181.2 (42.4)	171.9 (43.9)	200.4 (48.4)
Triglyceride (mg/dL)	106 (40.9)	78.4 (23.6)	84 (21.7)	85.6 (27.8)	103.2 (47.4)	95.5 (27.4)
HDL (mg/dL)	46.6 (17.4)	47.3 (15.2)	49.4 (15.2)	61.4 (15.1)	57.1 (11.4)	66.3 (19.1)
LDL (mg/dL)	93.1 (22.7)	86.7 (21.9)	88.9 (21.5)	104.9 (37.3)	96.4 (40.1)	118 (43.4)
ApoA1 (mg/dL)	140.6 (21.2)	145.4 (25.2)	147.6 (25.8)	172 (29.6)	163.4 (21.4)	190.6 (35.6)
ApoB (mg/dL)	78.4 (14.5)	82.3 (14.1)	79.4 (12.7)	87.2 (25.3)	82.4 (29.4)	92.3 (28.8)
CRP (mg/dL)	1.8 (2.2)	2.8 (3.0)	2 (1.7)	1.8 (1.1)	1.5 (1.0)	1.5 (1.4)
TSH (µIU/mL)	2 (0.75)	2.4 (0.95)	1.64 (0.71)	2.5 (0.9)	2.4 (1.0)	2.0 (0.9)
fT3 (ng/dL)	99.4 (16.0)	88.4 (16.0)	83.1 (9.6)	94.6 (8.0)	96.8 (6.8)	87.3 (13.3)
Cortisol (µg/dL)	14 (2.8)	14.2 (3.7)	10.7 (3.6)	10.5 (3.0)	11.8 (4.0)	10.5 (2.9)

Total cholesterol, triglyceride, HDL, LDL, ApoA1, ApoB, glucose, insulin, CRP*, TSH, fT3, cortisol inpatient day 1, inpatient day 7 and Week 3 KD and SD groups. Listed mean (Standard deviation). One participant with CRP elevation due to DVT/PE in KD group at week 3 excluded.

*CRP* C reactive protein, *TSH* Thyroid stimulating hormone ApoA1 and *ApoB* Apolipoprotein A1 and B.

*HDL* High-density lipoprotein, *LDL* Low-density lipoprotein.

In cognitive tests, both groups improved to a similar extent on the 3-back test % correct and 3-back/Stroop reaction time parameters from inpatient days 1 to 7 and week 3. The cognitive data are shown in an additional file [see Additional Tables 5-6].

The UPDRS scores showed a non-significant trend to improve from screening to week 3. For the UPDRS-1 in the KD group, there was a change from 4.6 ± 2.5 to 4.0 ± 2.3. There was also a trend for improvement in UPDRS-2 off scores in both groups during week 3 (KD 11.4 ± 3.1 to 10.0 ± 4.7; SD 14.2 ± 5.3 to 13.5 ± 5.0). No clear trends or statistically significant effects were observed in UPDRS-3 scores ([see Additional Table 7]).

Performance speed during the MN and PQ keyboard tasks improved for both groups ([see Additional Table 8]).

There was also improvement on the 9-hole pegboard test in the nondominant hand only in the KD group, 38.6 ± 11.2 s inpatient day 1, 33.1 ± 13.3 inpatient day 7, 33.1 ± 8.8 s week 3 [see Additional Table 9].

### Brain-derived neurotrophic factor

BDNF showed no significant effect of the intervention (KD inpatient day 1: 9.1 ± 3.1 ng/; inpatient day 7: 9.5 ± 3.9 ng/mL, week 3: 5.6 ± 4.9 ng/mL; SD inpatient day 1: 9.8 ± 5.4 ng/mL; inpatient day 7: 9.2 ± 4.8 ng/mL; week 3: 7.7 ± 4.7 ng/mL).

### EEG

Resting-state electroencephalography (rsEEG) showed a global decrease in relative delta power at inpatient day 7, more so in the KD group than in the SD group ([see Additional Table 10]). In addition, at inpatient day 7, there was an increase in relative beta power in both groups, globally in KD and frontal/parietal regions in SD. The relative fast (alpha1 + alpha2 + beta)/slow (delta + theta) band power increased in both groups at inpatient day 7, with a larger increase in power for the KD group. Changes at week 3 were attenuated, with a trend toward decreased delta power more posteriorly in both groups, a slight increase in global beta power in KD and parietal beta power in SD, and a mildly increased fast/slow power ratio globally in KD and frontal/parietal in SD.

rsEEG graph theory network connectivity measures showed that weighted kappa, a scalar connectivity measure of the sum of all weights of the edges indicating network strength, increased in the KD group (5.6 ± 3.7 inpatient day 1, 6.5 ± 3.9 inpatient day 7, 12.8 ± 11.2 week 3) as well as the SD group on day 7 but not at week 3 (5.7 ± 1.7 inpatient day 1, 12.2 ± 17.3 inpatient day 7, 7.3 ± 5.0 week 3). Similarly, the weighted clustering coefficient (weighted C, tendency of signal to organize in clusters) and mean clustering coefficient (CW_r) showed an increase at week 3 in the KD group compared with the SD group ([see Fig. 3, and tabulated results in Additional Table 11]).

**Fig. 4:**
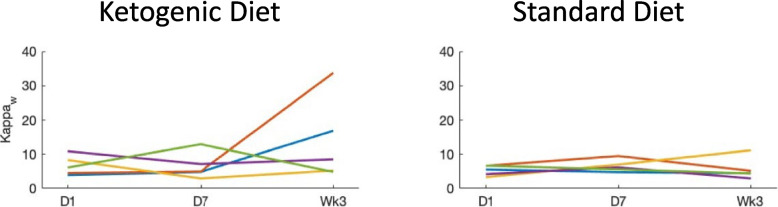
Resting-state EEG functional connectivity measure Kappa_w_ mean values per group KD (ketogenic diet). SD (standard diet) at preintervention (day 1), day 7, and week 3

Other connectivity measures (Rw, gamma, lambda, LW_r, weighted L, MST measures degree, eccentricity, betweenness centrality, Kappa, Diameter, leaf, mean) showed no significant differences (Fig. 4).

### Heart rate variability and orthostatic vital signs

Heart rate plethysmography time/frequency domains showed reduced variability in both groups on inpatient day 7. Otherwise, there were no clear differences across days of testing in either group [see Additional Table 12]. Similarly, HRV analysis from ECG electrodes using Kubios showed no clear trend as an effect of intervention in either group. Valsalva maneuver increased in the KD group: inpatient day 1 1.34 ± 0.10, inpatient day 7 1.47 ± 0.16, and week 3 1.41 ± 0.20, which was not seen in the SD group: inpatient day 1 1.35 ± 0.11, inpatient day 7 1.30 ± 0.10, and week 3 1.29 ± 0.12. The deep breathing inspiratory:expiratory ratio was similar between groups without the effect of intervention (Table 14).

**Table 14:** Heart Rate Variability

	Ketogenic Diet			Standard Diet		
	Day 1	Day 7	Week 3	Day 1	Day 7	Week 3
VM1 max	92.08 (3.95)	99.78 (7.05)	95.3 (9.37)	88.13 (7.79)	92.11 (9.59)	91.62 (16.9)
VM1 min	68.44 (7.19)	67.89 (7.47)	68.9 (6.42)	65.96 (5.11)	69.79 (5.64)	69.05 (7.99)
VR1	1.36 (0.14)	1.48 (0.19)	1.39 (0.17)	1.34 (0.15)	1.33 (0.1)	1.33 (0.17)
VM2 max	93.87 (3.87)	98.93 (4.93)	100.59 (22.12)	89.11 (7.47)	91.42 (12.13)	86.98 (11.21)
VM2 min	70.99 (6.18)	68.38 (6.64)	70.09 (8.4)	66.2 (7.18)	71.4 (7.23)	67.45 (8.84)
VR2	1.33 (0.11)	1.45 (0.12)	1.44 (0.26)	1.35 (0.09)	1.28 (0.11)	1.29 (0.12)
VR Avg	1.34 (0.1)	1.47 (0.16)	1.41 (0.2)	1.35 (0.11)	1.3 (0.1)	1.29 (0.12)
Inspiratory HR	73.94 (7.94)	80.14 (4.96)	75.87 (3.12)	73.29 (6.82)	78.66 (9.32)	74.82 (8.19)
Expiratory HR	68.78 (8.22)	72.96 (6.16)	70.2 (6.01)	67.54 (5.8)	71.16 (7.49)	69.2 (7.69)
E/I	1.08 (0.06)	1.1 (0.06)	1.09 (0.07)	1.09 (0.05)	1.11 (0.06)	1.08 (0.05)

*VM* Valsalva Maneuver (bpm), *VR* Valsalva Ratio, *E/I* Expiratory:Inspiratory ratio.

Blood pressure orthostatic testing showed no clear effect from the intervention in either group (Table 15).

**Table 15:** Orthostatic systolic blood pressure and heart rate pre/post intervention

	**Screening**	**Week 3**
Supine sBP	123 (14)	124 (7)
Standing Immediate sBP	104 (11)	110 (14)
Standing 3' sBP	114 (12)	117 (16)
Supine HR	71 (8)	68 (8)
Standing Immediate HR	81 (10)	77 (7)
Standing 3' HR	79 (8)	85 (7)

*sBP* Systolic blood pressure

## Discussion

In this study, in patients with PD, a short-term MCT-supplemented KD was feasible in > 90% of participants, as evidenced by week 3 serum ketosis > 0.5 mM in 94% of participants. Using MCT oil supplementation to comprise approximately 25% of the dietary fats was associated with high adherence, with average net carbohydrate intake over 2 weeks at home estimated to be less than 10% of energy intake, and the study dropout rate (1/16) was lower than in similar previous studies in PD.

Using magnetic resonance spectroscopy in healthy subjects, brain (occipital lobe) beta-OHB ketone values correlated well with plasma ketosis values (*r* = 0.86) with a brain-plasma slope of 0.26 [53]. Experimentally, the pathway of ketone body (primarily beta-OHB but also acetoacetate) entry across the blood brain barrier has been described via active transport (MCT1/MCT2) as well as diffusion. Plasma ketone values therefore appear to be a valid proxy for brain values.

There was no significant between-groups effect on mobility per TUG or UPDRS-3 at either inpatient day 7 or week 3; thus, the study was stopped for futility per the interim analysis. However, trends toward motor improvement were seen in the 9-hole pegboard and keyboard motor tasks. In rats administered a KD, anticipatory reward associated with phasic dopamine release was higher than that in rats fed regular CHO-containing chow despite no differences in striatal biochemical dopamine content, suggesting that diet-related physiological effects may differentially increase dopamine production and release (Pawlosky et al. unpublished data). One explanation for why we do not see a significant motor improvement is that the duration of the intervention may have been too short to see neuroplastic changes, such as those reported in an exercise intervention with increased posterior putamen/frontal connectivity [54]. Accordingly, improving the motor deficit of PD may require the combined effects of network strengthening, such as adding levodopa or ketones, and rewiring faulty synapses by using other interventions, such as rehabilitation or exercise.

Another possible reason for a lack of motor benefit could be that ketone metabolism more directly relates to nonmotor symptoms such as fatigue, somnolence, and anxiety. Such an interpretation is supported by this study, with the caveat that nonmotor symptoms were defined imprecisely via directed recall and self-report. This study’s finding of symptom reduction per nonmotor symptom scale, Gl rating scale, and exit survey free text in symptoms such as fatigue, mood, and wearing off/motor fluctuations were consistent with those previously reported using the KD in PD (Vanitallie et al. 2005 [25], Phillips et al. 2018 [26]). An exploratory outcome of cognition (Stroop effect) increased in the KD group within 6 days of starting the KD. This may indicate cognitive benefit similar to other studies that were attributed to ketosis [20] and supports future study of a KD, modified such as in this study with MCT supplement for ease of preparation and adherence, on the natural history of cognitive decline in PD. The trend of elevation in the L-DOPA metabolites dopamine and DOPAC in the KD group after 2 days of a ketogenic diet suggests that peripheral levodopa decarboxylase activity may be enhanced, possibly through changes in microbiota such as *Enterococcus faecalis*, which was found to convert levodopa to dopamine [55]. The microbiome is associated with disease and dietary patterns [56], although further study is needed.

Metabolic effects were consistent with those established in the literature and included weight loss, reduced waist circumference, elevated HDL, reduced TG, and improved insulin sensitivity [57, 58]. An elevated TG:HDL ratio is associated with coronary artery disease [59], and conversely, health benefits of carbohydrate/sugar reduction, including lowering the TG:HDL ratio, may also be seen. It is known that less glycated, less dense lipoproteins are less atherogenic. In this study, there were greater metabolic effects on reducing insulin and triglycerides and raising HDL seen in the KD group. This suggests a dose response of these metabolic parameters in the intensity of ketosis intervention. Participants without insulin resistance or metabolic syndrome showed a higher elevation in plasma ketones, a difference that could impact the longitudinal outcome. A significant reduction using bioelectric impedance spectroscopy read as skeletal mass was likely due to misallocation of water weight, as acute diuresis is an established effect of a KD [60]. The modest reduction in resting energy expenditure in PD from 2–3 weeks of a KD of approximately 100 kcal is a novel finding. This may be interesting to track in a future study to assess whether it is correlated with PD symptoms, as, for instance, a prior study showed an elevated metabolic rate in the postural instability/gait disorder subtype [61].

EEG showed increased global relative beta + alpha/theta + delta power in both groups but more in KD, consistent with a prior report describing KD in healthy volunteers, where a paired-pulse transcranial magnetic stimulation study over 2 weeks on the diet showed increased short intracortical inhibition (SICI), a phenomenon widely attributed to GABA-A signaling as well as increased central beta power on EEG [42]. Of potential relevance to PD, GABA is one of several deficient neurotransmitters due to selective neurodegeneration both in the striatum and in other sites in transgenic mice, including nuclei regulating REM onset, the preoptic nucleus and the nucleus reticularis [62]. In line with EEG connectivity changes observed in this study of increased Kappw and weighted C connectivity measures in the KD group compared to the SD group at Day 7 and Week 3, a similarly acute KD study in healthy volunteers showed increased network stability related to increased modularity of hubs [19], which could be related to dynamic effects of dopamine or GABA-mediated signaling. One possible mechanism of symptomatic benefit from a KD is elevation in dopamine signaling from nicotinamide adenine dinucleotide phosphate (NADPH) as a cofactor for tetrahydrobiopterin (BH4)-dependent dopamine synthesis. Conversely, carbohydrate and/or protein overfeeding raises blood glucose and insulin release and lipogenesis and depletes NADPH due to reduced citrate availability.

This study had several limitations. Negative findings of lack of improvement in TUG time and UPDRS motor score must be interpreted cautiously, as the ability to show improvement in these measures may have been limited by subjects with motor symptoms already well controlled by their other treatments, as ratings were performed in the ‘on’ dopaminergic medication state in all subjects and DBS ‘on’ stimulation state in 5/16 subjects. The SD group may have had more severe disease based on modestly higher daily levodopa daily equivalent dose despite shorter mean disease duration of 10 years (vs. 15 years in KD group) with higher number with DBS of 4 participants (vs. 2 in KD group). Limitations further include the small study size and short duration. There were differences in the composition of the inpatient and outpatient ketogenic diets with the aim of prioritizing diet satisfaction and adherence at home, and this may have affected feasibility or other study measures, though both diets consisted of the same macronutrient ratios and use of MCT oil. The feasibility of this protocol on a larger scale may depend on the availability of nutritionists/dietitians to assess outpatient adherence, although a less intensive method of ascribing to a scheduled menu combined with ketosis tracking has also been demonstrated [26]. Plasma BDNF has been proposed as a biomarker of PD [63] and has been shown to be decreased in PD patients with and without depression compared to control cohorts [64]. It was increased in the plasma of healthy volunteers following 4 weeks of a low-carb Paleolithic diet [65]. Although there was no correlation of plasma BDNF values to plasma BHB values in this study, that could have been simply a methodological limitation, from variabilities in clotting time or ELISA inaccuracy, and requires validation in further study.

## Conclusions

This study suggests that a KD with emulsified MCT oil supplementation is a feasible and acceptable intervention, however does not provide difference in motor score or mobility as tested in this three week study. While this study met its primary endpoint of feasibility, further study would be required to test the hypothesis that a modified KD incorporating MCT oil, such as that studied here or other supplements, would improve cognition and slow disease progression, ideally with the inclusion of reliable, accessible biomarkers such as resting-state EEG.

### Supplementary Information

**Supplementary Material 1. **

## Data Availability

The data supporting the findings of this study, including the full trial protocol are available on request from the corresponding author. The data are not publicly available due to privacy or ethical restrictions. The study is registered on ClinicalTrials.gov [63].
