# Supplementary material for: A randomized feasibility trial of medium chain triglyceride-supplemented ketogenic diet in people with Parkinson's disease
Source: BMC Neurol. 2024 Apr 1;24:106. doi: 10.1186/s12883-024-03603-5 (PMC10983636; doi:10.1186/s12883-024-03603-5)
Supplement: Supplementary file 1 — Supplementary Material 1. [file 12883_2024_3603_MOESM1_ESM.docx]

**Additional Files**

File name: Additional Table 1

File format: .pdf

Title: 24 hour recall net carb fractions in 2 weeks outpatient ketogenic diet

Description

File name: Additional Table 2

File format: .pdf

Title: 24 hour recall net carb fractions in 2 weeks outpatient ketogenic diet

Description: List of net carbohydrate energy fractions during the 2 weeks outpatient ketogenic diet for participants (left hand column in the inpatient ketogenic diet group and right hand column in the inpatient standard diet group).

File name: Additional Table 3

File format: pdf

Title: Ketogenic Diet Inpatient, Ketogenic Diet Outpatient Exit Survey

Description: exit survey results with description of benefits and side effects as applicable for each participant, and determination of likelihood of continuing a ketogenic diet such as tested in this study in the future, among the inpatient ketogenic diet group.

File name: Additional Table 4

File format: pdf

Title: Standard Diet Inpatient, Ketogenic Diet Outpatient Exit Survey

Description: exit survey results with description of benefits and side effects as applicable for each participant, and determination of likelihood of continuing a ketogenic diet such as tested in this study in the future, among the inpatient standard diet group.

File name: Additional Table 5

File format: pdf

Title: Cognitive Tests Ketogenic Diet

Description: Cognitive tests simple reaction time, complex reaction time, Stroop test, 3-back test results inpatient days 1-7 and week 3, among ketogenic diet group participants mean (standard deviation).

File name: Additional Table 6

File format: pdf

Title: Cognitive Tests Standard Diet

Description: Cognitive tests simple reaction time, complex reaction time, Stroop test, 3-back test results inpatient days 1-7 and week 3, among standard diet group participants mean (standard deviation).

File name: Additional Table 7

File format: pdf

Title: UPDRS

Description: UPDRS test results at screening visit and week 3 (parts 1-4) and also days 1-7 (part 3), divided by ketogenic diet and standard diet group participants.

File name: Additional Table 8

File format: pdf

Title: Keyboard MN and PQ tapping speeds

Description: Keyboard tapping speeds on alternating ‘M’-‘N’ and ‘P’-‘Q’ keys, latter using a single finger, on each side days 1-3 and week 3 visits, for ketogenic diet and standard diet groups. Listed values are mean (standard deviation).

File name: Additional Table 9

File format: pdf

Title: 9-hole pegboard test

Description: 9-hole pegboard test times (seconds) for ketogenic diet (KD) and standard diet (SD) groups days 1-7 and week 3 visits.

File name: Additional Table 10

File format: pdf

Title: EEG spectral power

Description: Values for Brainwave spectral power derivations from resting state EEG scans on day 1, day 7, and week 3 visits for ketogenic diet (KD) and standard diet (SD) groups, which were regionally defined (frontal F5 F3 F1 / Fz / F2 F4 F6; Central C5, C3, C1 / Cz / C2, C4, C6; Parietal P5 P3 P1 / Pz / P2 P4 P6) relative delta, relative beta, and fast:slow ratio (beta + alpha1 + alpha2)/(theta + delta).

File name: Additional Table 11

File format: pdf

Title: EEG connectivity

Description: Connectivity measures using graph theory network derivations from Brainwave software across EEG scan visits day 1, day 7, and week 3 for ketogenic diet (KD) and standard diet (SD) groups, which consisted of weighted mean clustering (Weighted C), weighted mean path length (Weighted L), weighted clustering coefficient (Cw_r), weighted length coefficient (Lw_r), and minimum spanning tree characteristics: gamma distribution (gamma), lambda distribution (lambda), random walk (Rw), weighted kappa (Kappa w), and weighted hub (Hw).

File name: Additional Table 12

File format: pdf

Title: 2-min heart rate variability

Description: 2-minute heart rate variability recording using EliteHrv photoplethysmography, ketogenic diet (KD) and standard diet (SD) groups days 1-7 and week 3. Performed in seated resting conditions.

File name: Additional Figure 1

File format: pdf

Title: Baseline and Week 3 Reported Exercise Levels

Description: . Survey results from weekly recall of exercise and activities at baseline (1 week prior to screening) and at week 3 visits, Metabolic equivalents * minutes/week 3, in ketogenic diet (KD) and standard diet groups (SD).

**Additional Table 1** Outpatient Ketogenic diet (KD) cookbook table of contents


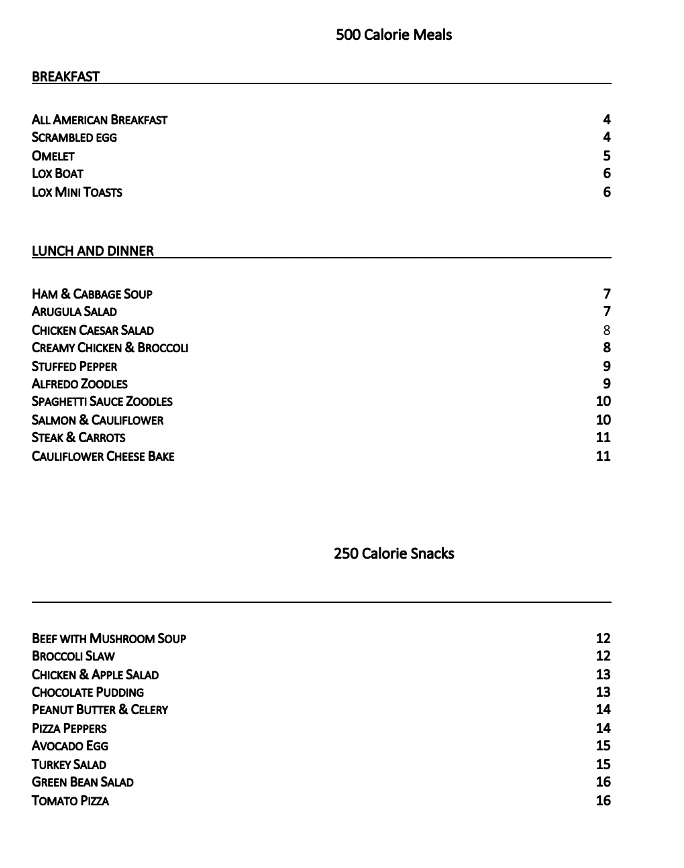


Outpatient Ketogenic diet (KD) cookbook table of contents. Each recipe met the same macronutrient requirements as the inpatient KD. Prior to inpatient discharge, participants received education and instructions from a Registered Dietitian Nutritionist (RDN) on following the diet at home. Based on participant’s individual calorie needs, RDNs prescribed a targeted number of 500 calorie meals and 250 calorie snacks from a KD cookbook specifically developed for this study. Each meal/snack recipe included 2 tbsp (30 mL) of MCT oil (Liquigen) per meal and 4 tsp (20 mL) MCT oil per snack. Participants were provided with enough Liquigen for the 2-week outpatient diet phase.

**Additional Table 2** 24 hour recall net carb fractions in 2 weeks outpatient ketogenic diet

| Subject ID | Net carb per 24h recall | Avg net carb | Subject ID | Net carb per 24h recall | Avg net carb |
| --- | --- | --- | --- | --- | --- |
| Inpt Ketogenic, Outpt Ketogenic |  |  | **Inpt Standard, Outpt Ketogenic** |  |  |
| 1 | 0.06 | 0.08 | 5 | 0.10 | 0.12 |
|  | 0.07 |  |  | 0.17 |  |
|  | 0.10 |  |  | 0.09 |  |
|  | 0.08 |  | 6 | 0.16 | 0.14 |
|  |  |  |  | 0.14 |  |
| 2 | 0.09 | 0.10 |  | 0.13 |  |
|  | 0.11 |  | 7 | 0.07 | 0.08 |
|  | 0.09 |  |  | 0.09 |  |
|  | 0.10 |  |  | 0.09 |  |
| 9 | 0.06 | 0.07 | 8 | 0.08 | 0.11 |
|  | 0.07 |  |  | 0.08 |  |
|  | 0.08 |  |  | 0.16 |  |
|  | 0.06 |  |  | 0.13 |  |
| 11 | 0.05 | 0.05 | 10 | 0.13 | 0.11 |
|  | 0.06 |  |  | 0.10 |  |
|  | 0.06 |  |  | 0.08 |  |
| 14 | 0.09 | 0.12 |  | 0.10 |  |
|  | 0.10 |  | 12 | 0.05 | 0.07 |
|  | 0.13 |  |  | 0.10 |  |
|  | 0.17 |  |  | 0.07 |  |
| 15 | 0.10 | 0.11 | 16 | 0.07 | 0.07 |
|  | 0.11 |  |  | 0.06 |  |
|  | 0.14 |  |  | 0.06 |  |
|  | 0.10 |  |  | 0.07 |  |
| 17 | 0.14 | 0.12 | 20 | 0.10 | 0.10 |
|  | 0.11 |  |  | 0.10 |  |
|  |  |  |  | 0.10 |  |
|  |  |  |  | 0.09 |  |

Net carb per unannounced 24 hour diet recalls in the 2 week at-home ketogenic diet open label extension, separated by inpatient randomized group (ketogenic diet lefthand columns, standard diet righthand columns). Each recall and averages shown. 8/15 participants (4 in each group) met 10% or less net carb intake. Average net carb 9.7% across all participants (not shown).

**Additional Table 3** Ketogenic Diet Inpatient, Ketogenic Diet Outpatient Exit Survey

| Subject ID | Benefits | Side effects / symptoms | Exit Survey |
| --- | --- | --- | --- |
| 1 | Mental clarity - faster. Thought process seems somewhat faster. E.g. activities today, seemed faster (testing Stroop etc). Health benefit of less sugar. | Significant issue of cramping right neck / diaphragm / respiratory muscles peaked at day 4-5 of keto diet peaked night 6-7, then mostly subsided. Feeling fatigued more than normal, week 1, week 2 better 80ish% normal. Would have liked more diet protein. | Very unlikely |
| 2 | Weight loss (lost 6-8 pounds water weight, about half in the 7 days in hospital). Thought he was in keto group initially while inpatient, but due to worsened fatigue at home thought he was in Usual Diet. | Fatigue, muscle aches worsened (left hip usual pain worse) and lower back. He was taking less Aleve than usual, only 4-5x/2 weeks vs daily before. | Somewhat likely |
| 9 | felt more energy, at baseline low energy in afternoons and this stabilized on the diet, also less hunger, didn't feel hungry as often | N/A | Agree. Very likely |
| 11 | Less muscle cramps, therefore taking less Artane (taken for muscle cramps), dry eyes/blurry vision. Last dose was Sat. Less posturing in hand (less 'off' related dystonic dyskinesia), no muscle cramps in > 1 week. Rigidity mildly improved, subtle onset throughout 2 weeks. Less symptomatic on left side. The muscle cramps improved in the first few days of starting the diet. Tremor is unchanged. | One occasion of diarrhea on 7/15, fine the next day. Appetite reduced (this is a benefit), misses bread but enjoys food. Prep time was fine, implemented some grab and go (Keto yogurt at Target, keto energy bar). No fatigue, headache, light-headedness, weakness. | Agree. Very likely |
| 14 | Curbed appetite, sometimes feel more energetic after exercise | There was fatigue, intermittently - worse end of day, better AM and after exercise. | Agree. Very likely |
| 15 | he did lose weight and appreciated that | feeling off several times as described (urinary incontinence, feeling stuck on 3 occasions, and balance worse - balance is a baseline condition) | Unsure |
| 17 | Felt improved mood - continue to be involved with exercise and felt more to be involved to do so, also weight loss is a plus | No ketoinduction symptoms, no fatigue / lightheadedness, etc - just did not have a bowel movement yesterday July 14, small BM July 15. | Agree. Very likely |

Reported acceptability per participant, inpatient ketogenic diet group after 2 weeks ketogenic diet at home open label extension. Likert scale refers to response to question: what is the likelihood you will continue a ketogenic diet such as tested in this study in the future’ 0=very unlikely, 1=unsure, 2=somewhat likely, 3=Agree. Very likely. Mean score across participants was 2.1 (somewhat to very likely to continue the ketogenic diet).*

*Note: Subject 1 experienced serious adverse event of pulmonary embolism in the setting of pre-existing hypercoagulability, considered to be not related to study intervention.

**Additional Table 4** Standard Diet Inpatient, Ketogenic Diet Outpatient Exit Survey

| Subject ID | Benefits | Side effects / symptoms | Exit Survey |
| --- | --- | --- | --- |
| 5 | lost weight | N/A | Somewhat likely |
| 6 | appetite less, felt full and lost weight, maybe more energy – able to do more activities; awake for longer (awakened early in AM as per usual, and did not feel sleepy in the afternoon) | No side effects, but burdensome to prepare and follow | Somewhat likely |
| 7 | Somewhat better walking, due to balance – believes less off balance, less holding onto surrounding objects 10% but still has issue | No side effect – for the first week fatigued but that went away. Followed diet closely as prescribed. There was also increased urination / hydration, and constipation and these also normalized. Still bradykinesia/rigidity there. Plan modification add MCT oil / coconut oil. | Agree. Very likely |
| 8 | Reduced dose of Rytary, tended to skip a dose most days, 12 or 4pm, as she felt on and was dyskinetic, didn’t feel like more dose was needed. Friend commented that she appeared more focused during task (shopping, active day) | Fatigue throughout 2 weeks, fluctuated, also did feel indigestion in the night once, does get that sometimes | Unsure |
| 10 | emotional, decreased anxiety – feeling positive about impacting his health through diet | none – no side effects | Agree. Very likely |
| 12 | Diminished action tremor (e.g. pushing buttons on Apple Watch), and mild reduction in gait freezing both noticed after 2-3 days on the diet. | Reduction in energy level occurring at 1-2 days on the ketogenic diet – somewhat lessened in second week but stable, persistent to time of this visit, did not interfere with ADLs (kept his usual walking, etc) | Agree. Very likely |
| 16 | Less fatigue, sleep improved (waking up less at night – baseline 2-3x nighttime awakenings unrelated to urination), urinary frequency unchanged, tremor mostly unchanged, absence of calves cramping (started April-May 2021, few times per week intense pain wake up at night). The only levodopa responsive symptom on the Rytary has been anxiety, which has been improved throughout study duration. | No keto-flu (just minor fatigue, at baseline – didn’t seem more than baseline), no exercise intolerance, weakness, cramps, etc. | Agree. Very likely |
| 20 | Less hunger than baseline; Less difficulty swallowing, slept well, better than baseline (as this is a significant complaint of insomnia at baseline), hallucinations upon awakening in the night with nocturia did not happen; less bloating in the evening; dyskinesia seemed to be better. | No side effects at any point (no any fatigue, dizziness, malaise, pain). There was no expectation of other symptom benefit that was wanting/lacking, no disappointment. | Somewhat likely |

Reported acceptability per participant, inpatient standard diet group after 2 weeks ketogenic diet at home open label extension. Likert scale refers to response to question: what is the likelihood you will continue a ketogenic diet such as tested in this study in the future’ 0=very unlikely, 1=unsure, 2=somewhat likely, 3=Agree. Very likely. Mean score across participants was 2.4 (somewhat to very likely to continue the ketogenic diet).

**Additional Table 5** Cognitive Tests Ketogenic Diet

| KETOGENIC | SRT | CRT | Stroop Congruent | Stroop Incongruent | Stroop Effect | Stroop % correct Congruent | Stroop % correct incongruent | Stroop % correct overall | 3-back RT | 3-back overall correct |
| --- | --- | --- | --- | --- | --- | --- | --- | --- | --- | --- |
| Day 1 | 365.53 (46.47) | 762.47 (368.35) | 1281.67 (262.85) | 1415.7 (261.97) | 133.94 (169.10) | 89.76 (12.24) | 81.73 (14.67) | 83.57 (12.24) | 1123.01 (236.73) | 68.57 (14.92) |
| Day 2 | 418.87 (98.26) | 732.99 (247.47) | 1236.57 (328.13) | 1332.76 (232.26) | 96.19 (215.77) | 86.83 (19.06) | 81.7 (17.31) | 83 (15.73) | 1043.56 (250.29) | 69.29 (18.13) |
| Day 3 | 368.33 (56.09) | 746.7 (390.63) | 1104.71 (184.46) | 1282.57 (272.93) | 185.94 (140.70) | 100 (0) | 87.7 (18.66) | 89.29 (17.48) | 977.33 (248.18) | 70.71 (15.92) |
| Day 4 | 425.64 (172.09) | 706.81 (301.93) | 1108.71 (222.25) | 1248.96 (225.19) | 152.27 (93.52) | 92.57 (16.04) | 92.74 (8.18) | 93.21 (7.32) | 972.2 (277.77) | 77.14 (19.55) |
| Day 5 | 412.13 (207.11) | 734.91 (428.42) | 1073.77 (263.21) | 1252.76 (247.66) | 161.84 (114.21) | 95.96 (7.69) | 91.9 (11.57) | 93.21 (9.54) | 952.07 (171.79) | 75 (18.48) |
| Day 6 | 387.97 (121.82) | 727.4 (332.35) | 1086.44 (242.58) | 1212.61 (254.75) | 126.23 (168.90) | 100 (0) | 91.49 (6.57) | 93.57 (4.30) | 841.44 (250.60) | 83.57 (17.96) |
| Day 7 | 432.93 (181.67) | 762.39 (408.12) | 1049.09 (159.57) | 1260.47 (223.87) | 197.1 (114.18) | 97.47 (4.37) | 91.84 (11.05) | 93.21 (9.21) | 958.06 (332.66) | 75.71 (23.35) |
| Wk 3 | 414.1 (164.97) | 778 (396.44) | 1133.27 (252.47) | 1260.24 (294.54) | 128.4 (182.91) | 98.41 (4.2) | 89.46 (11.44) | 91.79 (8.5) | 1001.27 (180.22) | 76.43 (15.74) |

**Additional Table 6** Cognitive Tests Standard Diet

| STANDARD DIET | SRT | CRT | Stroop Congruent | Stroop Incongruent | Stroop Effect | Stroop % correct Congruent | Stroop % correct incongruent | Stroop % correct overall | 3-back RT | 3-back overall correct |
| --- | --- | --- | --- | --- | --- | --- | --- | --- | --- | --- |
| Day 1 | 393.09 (77.53) | 847.2 (433.13) | 1253.11 (218.47) | 1380.28 (164.95) | 126.99 (137.56) | 89.38 (14.22) | 73.33 (26.66) | 77.78 (24.67) | 1119.08 (347.56) | 63.89 (22.19) |
| Day 2 | 412.76 (64.07) | 686.39 (101.74) | 1162.04 (295.90) | 1363.72 (286.16) | 201.68 (107.83) | 88.71 (28.12) | 81.23 (31.56) | 83.67 (30.96) | 1018.74 (388.76) | 68.89 (23.95) |
| Day 3 | 428.97 (106.54) | 676.14 (95.00) | 992.69 (312.77) | 1118.52 (322.18) | 125.81 (119.28) | 88.78 (28.70) | 86.99 (27.19) | 87.5 (27.64) | 982.96 (298.37) | 71.67 (14.58) |
| Day 4 | 381.91 (41.05) | 602.73 (79.95) | 1034.2 (137.67) | 1158.37 (97.89) | 124.17 (82.02) | 96.33 (7.97) | 88.28 (29.04) | 90.83 (22.01) | 853.12 (215.40) | 79.44 (10.14) |
| Day 5 | 412.29 (89.58) | 602.96 (60.90) | 1049.98 (238.34) | 1143.13 (153.03) | 93.12 (156.21) | 96.22 (11.33) | 86.37 (28.68) | 88.33 (25.53) | 818.37 (179.39) | 79.44 (10.14) |
| Day 6 | 411.14 (90.22) | 630.13 (96.72) | 1095.32 (295.01) | 1193.77 (228.51) | 98.44 (85.60) | 92.11 (23.67) | 87.29 (31.65) | 88.39 (30.36) | 908.78 (179.45) | 78.89 (9.28) |
| Day 7 | 396.32 (55.38) | 630.11 (75.86) | 955.1 (114.89) | 1121.66 (91.69) | 166.4 (86.06) | 91.7 (22.91) | 87.27 (28.70) | 88.61 (26.75) | 822.34 (251.52) | 78.89 (9.28) |
| Wk 3 | 377.68 (51.36) | 641.81 (82.08) | 1082.1 (154.81) | 1226.33 (166.79) | 144.24 (142.07) | 92.5 (20.13) | 84.4 (32.44) | 86.81 (28.58) | 936.55 (217.83) | 77.69 (15.10) |

**Additional Table 7** UPDRS

| **Visit** | **KD UPDRS 1** | **KD UPDRS 2 off** | **KD UPDRS 2 on** | **KD UPDRS 3** | **KD UPDRS 4** |
| --- | --- | --- | --- | --- | --- |
| Screening Visit | 4.6 (2.5) | 11.4 (3.1) | 8.3 (3.6) | 24.7 (8.1) | 3.9 (1.6) |
| Inpatient Day 1 |  |  |  | 24.4 (5.9) |  |
| Inpatient Day 2 |  |  |  | 25.6 (6.8) |  |
| Inpatient Day 3 |  |  |  | 24.3 (4.4) |  |
| Inpatient Day 4 |  |  |  | 24.7 (5.8) |  |
| Inpatient Day 5 |  |  |  | 23.6 (4.4) |  |
| Inpatient Day 6 |  |  |  | 23.4 (5.9) |  |
| Inpatient Day 7 |  |  |  | 24.6 (6.1) |  |
| Wk 3 | 4.0 (2.3) | 10.0 (4.7) | 7.3 (4.2) | 22.3 (4.5) | 3.3 (1.1) |
|  |  |  |  |  |  |
| **Visit Type** | **SD UPDRS 1** | **SD UPDRS 2 off** | **SD UPDRS 2 on** | **SD UPDRS 3** | **SD UPDRS 4** |
|  |  |  |  |  |  |
| Screening Visit | 3.7 (2.5) | 14.2 (5.3) | 10.7 (5.0) | 26.4 (6.9) | 5.9 (2.8) |
| Inpatient Day 1 |  |  |  | 25.2 (6.4) |  |
| Inpatient Day 2 |  |  |  | 26.6 (7.7) |  |
| Inpatient Day 3 |  |  |  | 27 (7.4) |  |
| Inpatient Day 4 |  |  |  | 26.2 (7.8) |  |
| Inpatient Day 5 |  |  |  | 26.8 (9.4) |  |
| Inpatient Day 6 |  |  |  | 23.2 (12.0) |  |
| Inpatient Day 7 |  |  |  | 24.1 (7.3) |  |
| Wk 3 | 4.1 (2.2) | 13.5 (5.0) | 10.4 (3.8) | 24.9 (8.2) | 5.0 (2.3) |

Section scores for parts of the Unified Parkinson’s Disease Rating Scale (UPDRS). Listed mean (Standard deviation). Complete UPDRS completed only on screening visit and week 3 visit. UPDRS part 3 was performed during all inpatient days 1-7.

**Additional Table 8** Keyboard MN and PQ tapping speeds

|  | KD MN total R | KD MN total L | KD MN k/s R | KD MN k/s L | KD PQ k/s R | KD PQ k/s L |
| --- | --- | --- | --- | --- | --- | --- |
| Admission Day 1 | 104 (36.38) | 92.29 (41.7) | 3.57 (1.27) | 3.18 (1.36) | 1.54 (0.44) | 1.47 (0.38) |
| Day 2 | 103.14 (37.64) | 95.43 (40.53) | 3.53 (1.27) | 3.36 (1.32) | 1.56 (0.31) | 1.36 (0.37) |
| Day 3 | 114.14 (49.09) | 90.29 (45.82) | 3.89 (1.64) | 3.07 (1.55) | 1.62 (0.31) | 1.54 (0.51) |
| Day 4 | 116.14 (40.49) | 96.86 (41.97) | 3.94 (1.42) | 3.34 (1.45) | 1.63 (0.29) | 1.53 (0.36) |
| Day 5 | 117 (50.13) | 101.71 (39.59) | 3.96 (1.69) | 3.45 (1.32) | 1.61 (0.36) | 1.41 (0.37) |
| Day 6 | 116 (51.96) | 95 (38.54) | 3.94 (1.76) | 3.25 (1.3) | 1.55 (0.44) | 1.56 (0.41) |
| Day 7 | 118.57 (50.06) | 96.57 (42.53) | 4.05 (1.72) | 3.28 (1.42) | 1.68 (0.35) | 1.64 (0.41) |
| Final visit Week 3 | 128 (58.39) | 99.43 (45.66) | 4.32 (1.93) | 3.41 (1.47) | 1.73 (0.42) | 1.51 (0.37) |
|  |  |  |  |  |  |  |
|  | **SD MN total, R** | **SD MN total, L** | **SD MN key/s, R** | **SD MN key/s, L** | **SD PQ key/s R** | **SD PQ key/s L** |
| Admission Day 1 | 101.25 (30.69) | 96.5 (38.86) | 3.45 (1.06) | 3.3 (1.31) | 1.51 (0.38) | 1.53 (0.4) |
| Day 2 | 98 (39.09) | 92.25 (39.09) | 3.34 (1.16) | 3.15 (1.31) | 1.51 (0.33) | 1.55 (0.32) |
| Day 3 | 99.75 (33.39) | 94 (33.39) | 3.41 (1.55) | 3.19 (1.15) | 1.62 (0.29) | 1.53 (0.56) |
| Day 4 | 118.13 (39.3) | 106.63 (39.3) | 4.02 (1.85) | 3.63 (1.3) | 1.59 (0.46) | 1.5 (0.42) |
| Day 5 | 113.63 (40.21) | 97.75 (40.21) | 3.88 (1.75) | 3.39 (1.3) | 1.55 (0.36) | 1.62 (0.45) |
| Day 6 | 111.38 (45.09) | 102.88 (45.09) | 3.8 (1.69) | 3.51 (1.52) | 1.56 (0.21) | 1.64 (0.3) |
| Day 7 | 120.5 (48.43) | 102.5 (48.43) | 4.12 (1.73) | 3.49 (1.61) | 1.61 (0.27) | 1.63 (0.43) |
| Final visit Week 3 | 109.29 (42.71) | 98.57 (42.71) | 3.72 (1.51) | 3.36 (1.44) | 1.66 (0.29) | 1.6 (0.49) |

Keyboard tapping MN total / 30s

**Additional Table 9** 9-hole pegboard test

|  | Ketogenic Diet | | Standard Diet | |
| --- | --- | --- | --- | --- |
|  | Dominant | Non-dominant | Dominant | Non-dominant |
| Day 1 | 29.1 (6.1) | 38.6 (11.2) | 27.9 (6.9) | 32.3 (7.4) |
| Day 2 | 34.3 (9.0) | 33.5 (10.9) | 29.1 (6.4) | 32.2 (5.9) |
| Day 3 | 29.7 (6.4) | 34.8 (12.0) | 26.7 (5.4) | 31.4 (6.0) |
| Day 4 | 32.0 (5.8) | 30.5 (5.7) | 26.6 (6.0) | 30.8 (6.5) |
| Day 5 | 30.6 (8.0) | 26.9 (15.1) | 27.8 (6.8) | 32.9 (7.4) |
| Day 6 | 30.1 (6.4) | 34.2 (13.0) | 27.9 (6.5) | 30.3 (8.9) |
| Day 7 | 31.0 (7.7) | 33.1 (13.3) | 26.4 (5.8) | 31.9 (10.5) |
| Week 3 | 29.4 (6.9) | 33.1 (8.8) | 27.7 (6.6) | 30.6 (5.9) |

Listed mean (Standard deviation)

**Additional Table 10**  EEG spectral power

|  | | Ketogenic Diet | | | Standard Diet | | |
| --- | --- | --- | --- | --- | --- | --- | --- |
|  |  | Frontal | Central | Parietal | Frontal | Central | Parietal |
| Day 1 | Delta power | 0.47 (0.22) | 0.36 (0.23) | 0.42 (0.27) | 0.46 (0.19) | 0.3 (0.12) | 0.35 (0.17) |
|  | Beta power | 0.15 (0.08) | 0.2 (0.12) | 0.16 (0.10) | 0.21 (0.07) | 0.29 (0.08) | 0.20 (0.07) |
|  | alpha1 + alpha2 + beta / theta + delta | 0.69 (0.53) | 1.14 (0.94) | 1.2  (1.24) | 0.88 (0.77) | 1.53 (0.85) | 1.56 (1.33) |
| Day 7 | Delta power | 0.31 (0.12) | 0.21 (0.07) | 0.2  (0.09) | 0.33 (0.21) | 0.25 (0.15) | 0.23 (0.19) |
|  | Beta power | 0.19 (0.12) | 0.26 (0.16) | 0.19 (0.08) | 0.24 (0.08) | 0.29 (0.06) | 0.25 (0.04) |
|  | alpha1 + alpha2 + beta / theta + delta | 1.37 (1.16) | 2.13 (1.84) | 2.81 (2.95) | 1.25 (0.73) | 1.74 (1.07) | 2.20 (1.68) |
| Week 3 | Delta power | 0.41 (0.25) | 0.33 (0.24) | 0.32 (0.27) | 0.4 (0.25) | 0.3 (0.20) | 0.29 (0.25) |
|  | Beta power | 0.17 (0.08) | 0.22 (0.11) | 0.19 (0.10) | 0.21 (0.10) | 0.27 (0.10) | 0.24 (0.10) |
|  | alpha1 + alpha2 + beta / theta + delta | 1.17 (1.22) | 1.39 (1.07) | 1.76 (1.78) | 1.13 (1.00) | 1.54 (0.95) | 1.90 (1.47) |

Resting state EEG relative spectral power, per defined regions Frontal, Central and Parietal, mean (standard deviation) in Ketogenic and Standard diet groups.

**Additional Table 11** EEG connectivity

|  | Ketogenic Diet | | | Standard Diet | | |
| --- | --- | --- | --- | --- | --- | --- |
|  | Day 1 | Day 7 | Week 3 | Day 1 | Day 7 | Week 3 |
| Weighted C | 0.1 (0.07) | 0.11 (0.07) | 0.21 (0.18) | 0.1 (0.03) | 0.21 (0.28) | 0.12 (0.08) |
| Weighted L | 66.4 (139.8) | 10.8 (4.4) | 7.2 (3.6) | 9.9 (3.3) | 6.7 (4.4) | 10 (5.0) |
| Cw_r | 0.08 (0.05) | 0.1 (0.06) | 0.2 (0.18) | 0.09 (0.02) | 0.19 (0.28) | 0.11 (0.08) |
| Lw_r | 59 (122.6) | 10.3 (5.00) | 6.8 (3.6) | 10.2 (3.5) | 6.4 (4.2) | 9.7 (5.0) |
| gamma | 1.18 (0.1) | 1.19 (0.09) | 1.08 (0.05) | 1.13 (0.06) | 4.6 (9.22) | 1.11 (0.06) |
| lambda | 1.06 (0.09) | 1.08 (0.1) | 1.08 (0.08) | 0.97 (0.05) | 0.92 (0.33) | 1.03 (0.04) |
| Rw | -0.09 (0.08) | -0.03 (0.05) | 0.01 (0.07) | -0.05 (0.04) | 0.06 (0.13) | -0.05 (0.05) |
| Kappa w | 5.66 (3.71) | 6.56 (3.87) | 12.78 (11.17) | 5.7 (1.7) | 12.21 (17.28) | 7.32 (5.05) |
| Hw | 0.76 (0.76) | 0.96 (1.12) | 4.42 (7.23) | 0.59 (0.32) | 6.73 (16.01) | 1.25 (1.57) |

**Additional Table 12** 2-min heart rate variability

| KETO |  | HR min | HR max | HR avg | RMSSD | lnRMSSD | SDNN | pnn50 | RR Mean | Total Power | LF power | LF peak | HF power | HF peak | LF/HF |
| --- | --- | --- | --- | --- | --- | --- | --- | --- | --- | --- | --- | --- | --- | --- | --- |
| Admission Day 1 |  | 66.6 | 77.9 | 71.6 | 26.3 | 2.9 | 22.6 | 7.6 | 844.9 | 351.6 | 128.6 | 0.1 | 224.9 | 0.5 | 0.6 |
| Day 2 |  | 70.1 | 77.9 | 73.6 | 18.4 | 2.7 | 16.1 | 3.6 | 820.4 | 96.8 | 41.7 | 0.1 | 55.1 | 0.3 | 1.4 |
| Day 3 |  | 69.6 | 80.3 | 74.3 | 23.7 | 8.3 | 18.9 | 2.4 | 818.2 | 113.1 | 46.4 | 0.1 | 66.5 | 0.3 | 0.7 |
| Day 4 |  | 72.4 | 81.7 | 76.6 | 17.8 | 2.6 | 26.6 | 1.7 | 786.0 | 206.6 | 106.1 | 0.1 | 100.6 | 0.3 | 1.4 |
| Day 5 |  | 73.0 | 82.0 | 76.9 | 21.7 | 2.9 | 17.9 | 6.0 | 785.2 | 135.2 | 55.1 | 0.1 | 80.1 | 0.3 | 0.9 |
| Day 6 |  | 73.4 | 83.3 | 77.7 | 14.2 | 3.9 | 13.6 | 0.7 | 777.0 | 74.6 | 36.4 | 0.1 | 38.3 | 0.3 | 1.1 |
| Day 7 |  | 73.0 | 82.0 | 76.9 | 14.8 | 2.6 | 16.1 | 1.9 | 784.1 | 143.0 | 75.8 | 0.1 | 67.2 | 0.3 | 2.3 |
| Final visit Week 3 |  | 66.4 | 81.4 | 72.6 | 23.0 | 3.0 | 23.8 | 4.7 | 835.2 | 278.1 | 86.0 | 0.1 | 192.1 | 0.3 | 1.0 |

| STANDARD | HRmin | HRmax | HR avg | RMSSD | lnRMSSD | SDNN | pnn50 | RR Mean | Total Power | LF power | LF peak | HF power | HF peak | LF/HF |
| --- | --- | --- | --- | --- | --- | --- | --- | --- | --- | --- | --- | --- | --- | --- |
| Admission Day 1 | 66.3 | 80.9 | 72.9 | 27.3 | 3.2 | 26.8 | 7.6 | 830.3 | 300.2 | 143.3 | 0.1 | 156.9 | 0.3 | 1.1 |
| Day 2 | 71.1 | 85.8 | 78.6 | 24.6 | 3.1 | 24.3 | 8.4 | 776.0 | 209.1 | 112.5 | 0.1 | 96.6 | 0.3 | 1.3 |
| Day 3 | 72.8 | 85.1 | 77.8 | 23.6 | 3.0 | 23.2 | 7.4 | 780.1 | 240.7 | 112.4 | 0.1 | 128.4 | 0.3 | 1.2 |
| Day 4 | 76.7 | 85.3 | 81.2 | 14.0 | 2.5 | 15.8 | 0.8 | 747.2 | 111.3 | 71.1 | 0.1 | 40.3 | 0.3 | 1.8 |
| Day 5 | 70.4 | 83.0 | 77.7 | 25.1 | 3.1 | 20.5 | 7.9 | 777.2 | 172.0 | 91.6 | 0.1 | 80.4 | 0.3 | 1.5 |
| Day 6 | 76.6 | 87.2 | 81.2 | 18.5 | 2.8 | 18.2 | 3.7 | 745.9 | 191.9 | 61.7 | 0.1 | 130.1 | 0.3 | 0.7 |
| Day 7 | 69.9 | 82.4 | 76.9 | 24.5 | 3.1 | 22.4 | 5.3 | 796.5 | 899.4 | 421.8 | 0.1 | 477.6 | 0.3 | 1.4 |
| Final visit Week 3 | 67.9 | 77.9 | 72.5 | 26.0 | 5.9 | 20.7 | 9.7 | 839.3 | 284.0 | 159.2 | 0.1 | 126.1 | 0.3 | 1.3 |

2-min heart rate variability measures using EliteHRV photoplethysmography in Ketogenic Diet (Keto) and Standard Diet groups, mean values. Final week 3 visit follows the same 2 week open label extension ketogenic diet at home for both groups.

**Additional Figure 1** Baseline and Week 3 Reported Exercise Levels

Exercise levels were converted to metabolic equivalents (MET) * minutes/week, based on survey data of the recalled activity type / duration and standard MET values from the 2011 Adult Compendium of Physical Activities, at baseline (screening, from the week prior to screening) and at week 3, in ketogenic diet (KD) and standard diet groups (SD) plotted by participant.
